# Brain morphometry in older adults with and without dementia using extremely rapid structural scans

**DOI:** 10.1016/j.neuroimage.2023.120173

**Published:** 2023-05-16

**Authors:** Maxwell L. Elliott, Lindsay C. Hanford, Aya Hamadeh, Tom Hilbert, Tobias Kober, Bradford C. Dickerson, Ross W. Mair, Mark C. Eldaief, Randy L. Buckner

**Affiliations:** a Department of Psychology, Center for Brain Science, Harvard University, 52 Oxford Street, Northwest Laboratory 280.10, Cambridge, MA 02138, USA; b Baylor College of Medicine, Houston, TX 77030, USA; c Advanced Clinical Imaging Technology, Siemens Healthineers International AG, Lausanne, Switzerland; d Department of Radiology, Lausanne University Hospital and University of Lausanne, Lausanne, Switzerland; e LTS5, École Polytechnique Fédérale de Lausanne (EPFL), Lausanne, Switzerland; f Frontotemporal Disorders Unit, Massachusetts General Hospital, USA; g Alzheimer’s Disease Research Center, Massachusetts General Hospital, USA; h Athinoula A. Martinos Center for Biomedical Imaging, Massachusetts General Hospital, USA; i Department of Neurology, Massachusetts General Hospital & Harvard Medical School, USA; j Department of Psychiatry, Massachusetts General Hospital & Harvard Medical School, Charlestown, MA 02129, USA

**Keywords:** MRI, Hippocampus, ADNI, Aging, Alzheimer’s disease, Frontotemporal lobar degeneration

## Abstract

T1-weighted structural MRI is widely used to measure brain morphometry (e.g., cortical thickness and subcortical volumes). Accelerated scans as fast as one minute or less are now available but it is unclear if they are adequate for quantitative morphometry. Here we compared the measurement properties of a widely adopted 1.0 mm resolution scan from the Alzheimer’s Disease Neuroimaging Initiative (ADNI = 5′12”) with two variants of highly accelerated 1.0 mm scans (compressed-sensing, CSx6 = 1′12”; and wave-controlled aliasing in parallel imaging, WAVEx9 = 1′09”) in a test-retest study of 37 older adults aged 54 to 86 (including 19 individuals diagnosed with a neurodegenerative dementia). Rapid scans produced highly reliable morphometric measures that largely matched the quality of morphometrics derived from the ADNI scan. Regions of lower reliability and relative divergence between ADNI and rapid scan alternatives tended to occur in midline regions and regions with susceptibility-induced artifacts. Critically, the rapid scans yielded morphometric measures similar to the ADNI scan in regions of high atrophy. The results converge to suggest that, for many current uses, extremely rapid scans can replace longer scans. As a final test, we explored the possibility of a 0′49” 1.2 mm CSx6 structural scan, which also showed promise. Rapid structural scans may benefit MRI studies by shortening the scan session and reducing cost, minimizing opportunity for movement, creating room for additional scan sequences, and allowing for the repetition of structural scans to increase precision of the estimates.

## 1. Introduction

Structural MRI is widely used to measure brain morphometry (e.g., measurements of global and regional brain volumes and cortical thickness). Individual differences in morphometric measures have been linked to aging, behavior, and brain disorders (Maguire et al., 2000; Raz et al., 2010; Schmaal et al., 2016; Smith et al., 2020; van Erp et al., 2016). For example, older adults tend to have a thinner cortex and smaller subcortical volumes than younger adults (Bethlehem et al., 2022; Douaud et al., 2014; Hogstrom et al., 2013; Raz et al., 2005; Salat et al., 2004). Furthermore, spatially distinct “signature” patterns of cortical atrophy are useful for diagnosis, prognostication, and longitudinal outcome monitoring in neurodegenerative disease (e.g., Alzheimer’s disease (AD) and Frontotemporal Lobar Degeneration (FTLD); Bejanin et al., 2020; Dickerson et al., 2009, 2011; Dickerson and Wolk, 2013; Frisoni et al., 2010; Jack et al., 2013; Johnson et al., 2012; Keret et al., 2021; Knopman et al., 2016). In addition to morphometric analyses, structural MRI is commonly used as a reference for functional MRI. In aggregate, structural MRI consumes extensive resources because a structural scan is collected in nearly every MRI session.

The current standard scan for brain morphometry is a 1.0 mm isotropic magnetization-prepared gradient echo (MPRAGE) acquisition that takes 4–8 min to collect on a 3T scanner with modest in-plane acceleration. SENSitivity Encoding (SENSE) and Generalized Autocalibrating Partially Parallel Acquisitions (GRAPPA) are two common in-plane acceleration techniques that reduce scan time by exploiting redundancies in the data collected across nearby sensors in multi-channel head coils (Griswold et al., 2002; Pruessmann et al., 1999). However, with GRAPPA and SENSE, acceleration past 2x in MPRAGE scans is limited because noise-amplification compounds as acceleration increases, leading to diminishing returns at higher levels of acceleration. Specifically, morphometric measurement problems arise, especially in sub-cortical structures, as signal-to-noise decreases.

Modest in-plane acceleration has been widely adopted for large efforts focused on morphometric investigations of brain aging such as the Alzheimer’s Disease Neuroimaging Initiative (ADNI; 1.0 mm isotropic MPRAGE, 5 – 7 min depending on the MRI system, 2x acceleration; Gunter et al., 2017; Jack et al., 2015) and the Human Connectome Project in Aging (HCP-A; 0.8 mm isotropic MPRAGE, 8′22”, 2x acceleration; Bookheimer et al., 2019). Data collection for the UK Biobank illustrates the limits of current in-plane acceleration techniques. The UK Biobank targets 100,000 participants. Due to this scale, each additional minute of scan time costs the study over one million dollars (Miller et al., 2016). To minimize costs and burden, the UK Biobank adopted a 1.0 mm isotropic MPRAGE using 2x acceleration and a tight field of view to achieve a 4′54” acquisition time (Miller et al., 2016).

Recent progress in scan acceleration has yielded two new promising techniques for pushing rapid scanning even further: compressed sensing (CS) and Wave controlled aliasing in parallel imaging (Wave-CAIPI). Both CS and Wave-CAIPI techniques can acquire MPRAGE images in less than 90 s with noise amplification that is comparable to standard MPRAGE images produced with GRAPPA and SENSE acceleration (Dieckmeyer et al., 2021; Mussard et al., 2020; Polak et al., 2018). While CS and Wave-CAIPI can achieve similar levels of acceleration, each accomplishes acceleration with a distinct methodological advance. CS is a general signal-processing technique that adopts advances in information theory to achieve data compression (Candes and Wakin, 2008). When applied to MPRAGE acquisitions, CS accelerates scans by using prior knowledge about the sparsity of the MRI signal to incoherently under-sample k-space. Then tailored algorithms allow for reconstruction with minimal noise amplification (Lustig et al., 2007; Yang et al., 2016). Wave-CAIPI is an MRI-specific acceleration tool that advances in-plane acceleration techniques by combining controlled aliasing in parallel imaging acceleration, along two dimensions using sinusoidal wave encoding, with corkscrew trajectories through k-space (Bilgic et al., 2015; Polak et al., 2018). While CS and Wave-CAIPI have both demonstrated substantial acceleration with tolerable noise amplification, their viability to replace standard longer scans for estimating morphometric measures is uncertain.

Here we investigate the reliability, precision, and convergent validity of morphometric measures derived from extremely rapid CS and Wave-CAIPI scans as compared to a field-standard contemporary MPRAGE protocol. Critically, we compared scan types in a sample of older adults that included individuals with neurodegenerative dementias. This sample was chosen because older adults, especially those with neurodegenerative disease, are a population that is of central interest to morphometric studies and one that will expose limitations in rapid scanning techniques due to the presence of challenges for automated morphometry including atrophy, reduced contrast, and head motion. Individuals with focal and often asymmetric atrophy due to AD or FTLD were enrolled allowing differences between techniques to be assessed in cases where local atrophy can be extreme and non-uniform (Collins et al., 2017). Morphometric measures from a reference structural scan based on the “ADNI-3 Advanced” protocol (1.0 mm 5′12” acquisition, 2x GRAPPA acceleration; referred to as ADNI hereafter) were compared to a CS acquisition (1.0 mm 1′12” acquisition with 6x acceleration; referred to as CSx6 hereafter) and a Wave-CAIPI acquisition (1.0 mm 1′09” acquisition with 3 × 3 acceleration; referred to as WAVEx9 hereafter). As the results will reveal, we found that rapid scans acquired in about a minute can replace longer scans for many morphometric applications.

## 2. Methods

### 2.1. Participants

Thirty-eight older participants were recruited from the Massachusetts Alzheimer’s Disease Research Center and the Frontotemporal Disorders Unit at the Massachusetts General Hospital. Participants were either cognitively unimpaired (Clinical Dementia Rating, CDR = 0; *n =* 18) or with very mild, mild, or moderate dementia (CDR = 0.5, 1 or 2) with a clinical diagnosis of either the temporal lobe variant of FTLD (*n =* 9), amnestic mild cognitive impairment (*n =* 4), Alzheimer’s dementia (*n =* 5) or uncertain MCI (*n =* 1). At the time of scanning, three of the FTLD participants’ presentations were semantic variant Primary Progressive Aphasia (svPPA; Gorno-Tempini et al., 2011), three had developed typical behavioral symptoms and would best be classified as Semantic Dementia (Neary et al., 1998), two participants’ presentations were behavioral variant Frontotemporal Dementia (Rascovsky et al., 2011), and one participant’s presentation was semantic-behavioral variant Frontotemporal Dementia (Younes et al., 2022). We chose this group of participants to explore the viability of rapid scans across individuals with distinct patterns and degrees of atrophy. All participants provided written informed consent in accordance with the guidelines of the Institutional Review Board of Mass General Brigham Healthcare and were compensated. CDR scores and CDR + NACC FTLD scores (Miyagawa et al., 2020) were obtained from recent clinical or research visits. Due to head motion and poor data quality detected during quality control, one participant (CDR = 0) was excluded from all analyses. This resulted in a final sample of 37 analyzed participants (20 females; 71.2 +/− 7.6 years; age range: 54 – 86 years; Table 1).

### 2.2. MRI data acquisition

MRI data were collected at the Harvard Center for Brain Science using a 3T Siemens MAGNETOM Prisma^fit^ MRI scanner (Siemens Healthcare; Erlangen, Germany) and the vendor’s 32-channel head coil. The scanner and ADNI protocols were certified via the Standardized Centralized Alzheimer’s & Related Dementias Neuroimaging (SCAN) initiative (https://scan.naccdata.org/). During the scanning sessions, participants were encouraged to remain still and given the option to listen to music or watch video clips (e.g., a nature documentary). Inflatable cushions were used to immobilize the participants’ heads. Every 5–10 min participants were given reminders to stay still and feedback about their level of motion.

The study protocol was designed to compare a standard three-dimensional T1-weighted MPRAGE from the ADNI protocol (Weiner et al., 2017), to rapid alternatives. Specifically, we compared the ADNI reference T1-weighted scan (Weiner et al., 2017) to two research application rapid T1-weighted sequences. The two rapid acceleration techniques were 1) CSx6 (Mussard et al., 2020) and 2) WAVEx9 (Polak et al., 2018). To estimate reliability, all participants completed two scanning sessions on separate days (i.e., test-retest) within a short period (mean time between scans = 8.2 days +/− 5.5 days; 1 – 25 days). Reliability was calculated by comparing measures for the same scan type acquired on two separate days (Session 1 versus Session 2). For analyses of validity, the ADNI scan was compared to the rapid alternative on the same day, allowing two separate estimates of validity (validity within Session 1 and validity within Session 2).

We investigated 5 different T1-weighted scans: (1) 1.0 mm isotropic ADNI MPRAGE acquisition (5′12” acquisition; pulse repetition time (TR) = 2300 ms; inversion time (TI) = 900 ms; time to echo (TE) = 2.98 ms; flip angle = 9°; field of view 256 × 240 × 208 mm; acquisition orientation = sagittal; in-plane GRAPPA acceleration = 2) (Weiner et al., 2017), (2) 1.0 mm isotropic CSx6 scans (1′12” acquisition; TR = 2300 ms; TI = 900 ms; TE = 2.96 ms; flip angle = 9°; field of view = 256 × 192 × 240 mm; acquisition orientation = coronal; compressed sensing acceleration = 6x), (3) 1.0 mm WAVEx9 scans (1′09” acquisition; TR = 2300 ms; TI = 900 ms; TE = 3.24 ms; flip angle = 9°; field of view = 256 × 240 × 192 mm; acquisition orientation = sagittal; Wave acceleration = 3 × 3), (4) 0.8 mm isotropic compressed-sensing scans (1′49” acquisition; TR = 2300 ms; TI = 900 ms; TE = 3.1 ms; flip angle = 9°; field of view = 256 × 192 × 230 mm; acquisition orientation = coronal; compressed sensing acceleration = 6x), and (5) 1.2 mm isotropic CSx6 scans (0′49” acquisition; TR = 2300 ms; TI = 900 ms; TE = 2.86 ms; flip angle = 9°; field of view = 230 × 194 × 230 mm; acquisition orientation = coronal; compressed sensing acceleration = 6x). Notably, we used a coronal acquisition for the CSx6 scans in this study in contrast to the sagittal acquisitions of the ADNI and WAVEx9 scans. We set the acquisition direction of the CSx6 scans to coronal after piloting revealed that the sagittal acquisition orientation compounded susceptibility-induced artifacts in the orbitofrontal cortex in CSx6 scans (Hanford et al., 2021).

Across participants, the order of the ADNI and rapid scans was counterbalanced to allow for head-to-head comparisons. This set of scans allowed for direct comparisons of each rapid scan acceleration method to the ADNI scan, holding voxel size (1.0 mm) constant and with scan order counterbalanced. Additionally, exploratory follow-up analyses investigated the 0.8 mm CSx6 scan and the 1.2 mm CSx6 scan.

### 2.3. Image processing and morphometry

All structural images were processed with FreeSurfer version 6.0.1 using the recon-all processing pipeline (Dale et al., 1999; Fischl et al., 1999). Each scan was processed independently of the others. The direct results from the automated recon-all pipeline were used without edits or manual interventions. Recon-all included volume-based processing and surfaced-based processing. Volume-based processing included intensity normalization, skull stripping (Ségonne et al., 2004), and segmentation of regional brain volumes (Fischl et al., 2002). Next, surfacebased processing generated a model of the white-matter surface and the pial surface from each scan (Dale et al., 1999; Fischl et al., 1999). Results were then used to estimate morphometric measures including global brain volumes and thickness measures, regional brain volumes, and regional cortical thickness measures from the Desikan-Killiany atlas (Desikan et al., 2006; Fischl et al., 2004).

All structural images were visually inspected to note motion artifacts, banding, ringing, and blurring. Visual inspection revealed minor banding artifacts in the CSx6 scans that were most evident in the coronal plane. While visually apparent in raw images, visual inspection of automated labeling and estimated pial and gray/white matter surfaces revealed that these minor artifacts did not visibly affect the estimation process for the CSx6 scans, an impression that was tested extensively in quantitative analyses. In addition, the results of the recon-all pipeline were checked to confirm that automated processing was completed without error.

### 2.5. Image quality metrics

Image quality metrics were calculated using the MRIQC software package (Esteban et al., 2017). Specifically, four widely used metrics of image quality were investigated, including: (1) the average signal-to-noise ratio in white matter voxels (SNR WM), (2) the average signal-to-noise ratio in gray matter voxels (SNR GM), (3) the contrast-to-noise ratio (CNR) - which is an estimate of how distinct the image intensities are between the distributions of gray matter and white matter voxels, and (4) the full-width at half maximum (FWHM) – which is a quantitative estimate of spatial smoothness.

### 2.6. Test-retest reliability and measurement error analyses

To explore the reliability and measurement precision of each morphometric measure derived from each scan type, two separate analyses were conducted. The first analysis explored test-retest reliability, and the second analysis explored measurement error.

We estimated the test-retest reliability for each scan type (i.e., ADNI, CSx6, and WAVEx9) across 87 separate morphometric measures. These included three global measures (estimated total intracranial volume, eTIV, Buckner et al., 2004; whole brain volume, WBV; and mean cortical thickness); 16 subcortical volumes (left and right estimates of the amygdala, accumbens / nucleus accumbens, pallidum / globus pallidus, caudate nucleus, hippocampus, putamen, thalamus, and ventral diencephalon volume from the Aseg atlas; Dale et al., 1999), and 68 regional cortical thickness measures (all cortical regions from the DesikanKilliany atlas; Desikan et al., 2006). Reliability was operationalized for each measure as the amount of Session 2 variance that could be explained by Session 1 variance using linear regression. All reliability estimates are reported as R^2^ coefficients from these linear regression models.

For each scan type and morphometric measure, we estimate the proportion of each measure that is due to measurement error (i.e., percent error). We estimated the percent error for each morphometric measure as the absolute difference between the estimated measure from Session 1 and Session 2 divided by the average total size of the measure. Larger values indicate greater divergence between estimates and a higher proportion of the measurement that is attributable to measurement error (i.e., lower precision). In addition to reporting percent errors in the main text, we also provide both absolute errors and percent errors for each morphometric and each scan type in the supplemental materials.

### 2.7. Convergent validity analyses

We estimated the validity of each morphometric measure by directly comparing estimates from the CSx6 and WAVEx9 scans to the ADNI scan. That is, we asked whether the rapid scan alternatives would capture the same between-subject variance captured by the ADNI scan.

For each morphometric measure, validity was operationalized as the amount of between-subject variance in each morphometric measure derived from the ADNI scans that could be explained by the corresponding morphometric measure derived from the rapid scans. All validity estimates are reported as R^2^ coefficients from these linear regression models. To estimate the stability of these validity estimates, the same procedure was used to estimate validity from each session independently, yielding two validity estimates.

In addition, we estimated the sensitivity of each rapid scan as the slope of the line of best fit from these convergent validity linear regression models. Slopes less than one indicate a restriction of range in the rapid scan and a potential loss of sensitivity relative to the ADNI scan. By contrast, a slope near the identity line (*X* = Y) and a high R^2^ coefficient indicates that the rapid scan largely captures the same information as the ADNI scan.

### 2.8. Visualization

**Fig. 1.**
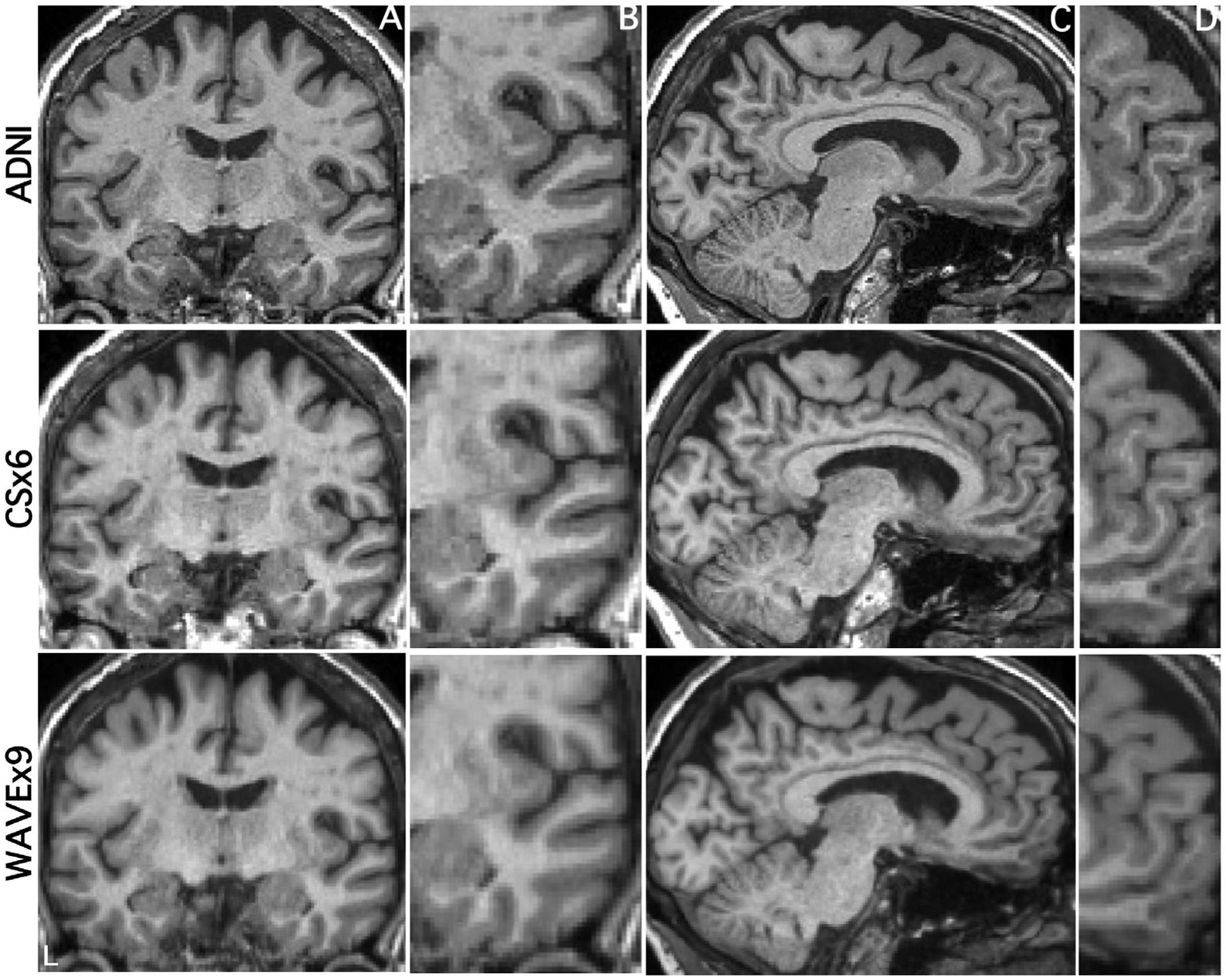
Extremely rapid compressed-sensing and Wave-CAIPI protocols generate morphometrics-ready high-resolution T1-weighted structural scans. The same coronal and sagittal slices (A, C) alongside a zoomed-in portion of each respective slice (B, D) are displayed from the same scan session for three scan types from a single representative participant (77-year-old cognitively unimpaired female). In row 1, images are from a standard 1.0 mm isotropic T1-weighted acquisition (5′12”; ADNI). In row 2, images are from the 1.0 mm isotropic CSx6 acquisition (1′12”). CSx6 achieves a ~5-fold reduction in scan time compared to the standard ADNI acquisition by sparsely sampling k-space data during acquisition. In row 3, images are from the 1.0 mm isotropic WAVEx9 acquisition (1′09”). WAVEx9 achieves a ~5-fold reduction in acquisition time through parallel imaging. Note that while the CSx6 and WAVEx9 achieve high-resolution images, there are differences in image quality when compared with the ADNI image. In B the boundary between the pallidum, putamen, and the surrounding white matter shows lower contrast. In D, the medial prefrontal cortex appears grainier with lower contrast.

**Fig. 2.**
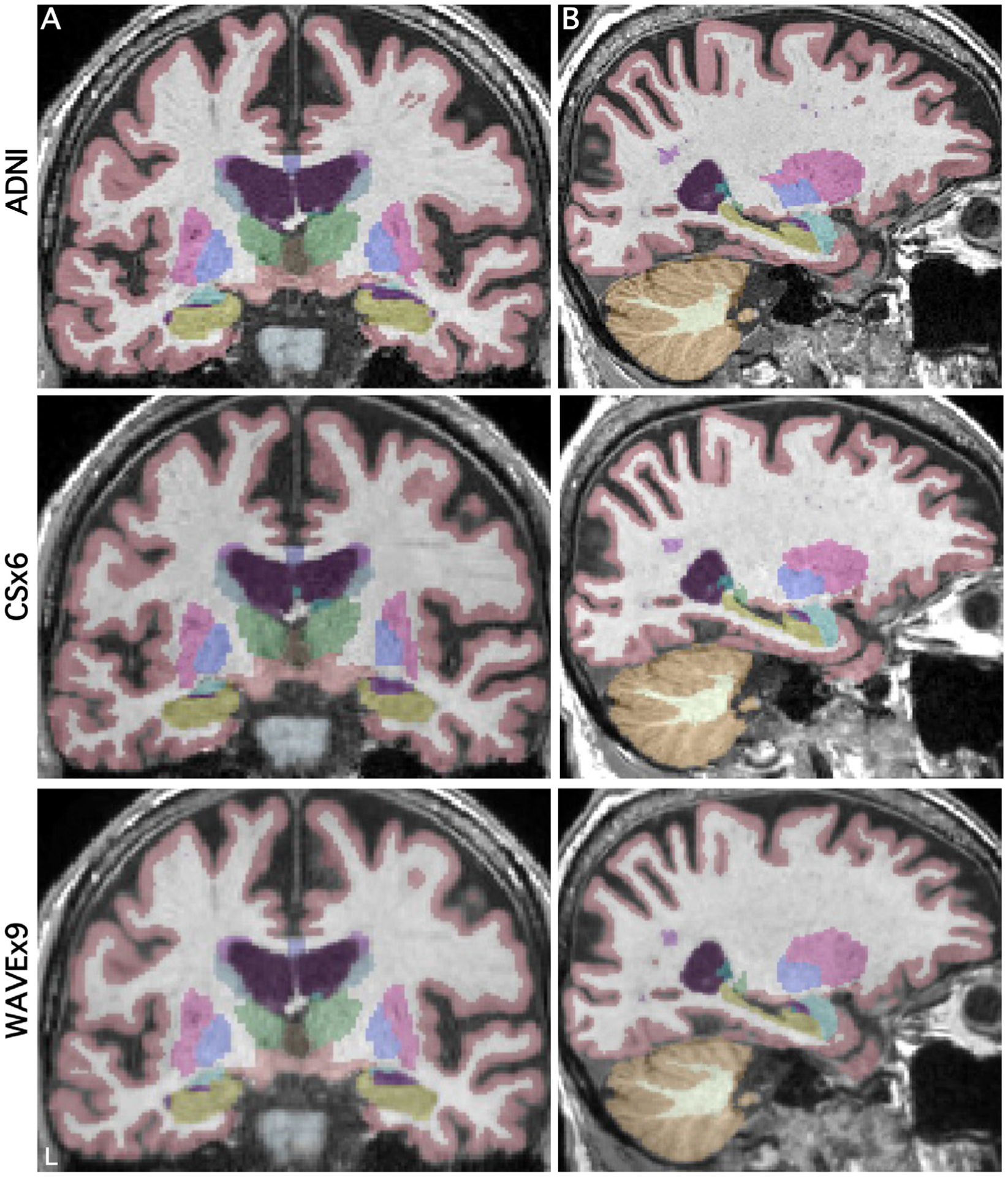
Parcellations of subcortical structures can be estimated from rapid structural scans. Automated volumetric labeling is illustrated from FreeSurfer’s recon-all pipeline for the ADNI (row 1), CSx6 (row 2), and WAVEx9 (row 3) images. To aid visual comparison, coronal (A) and sagittal (B) sections from each scan type from a single representative participant are shown (86-year-old cognitively unimpaired male). Volumetric labels (FreeSurfer aseg) are successfully estimated in the rapid scans and are comparable to the ADNI scan with only minor differences.

**Fig. 3.**
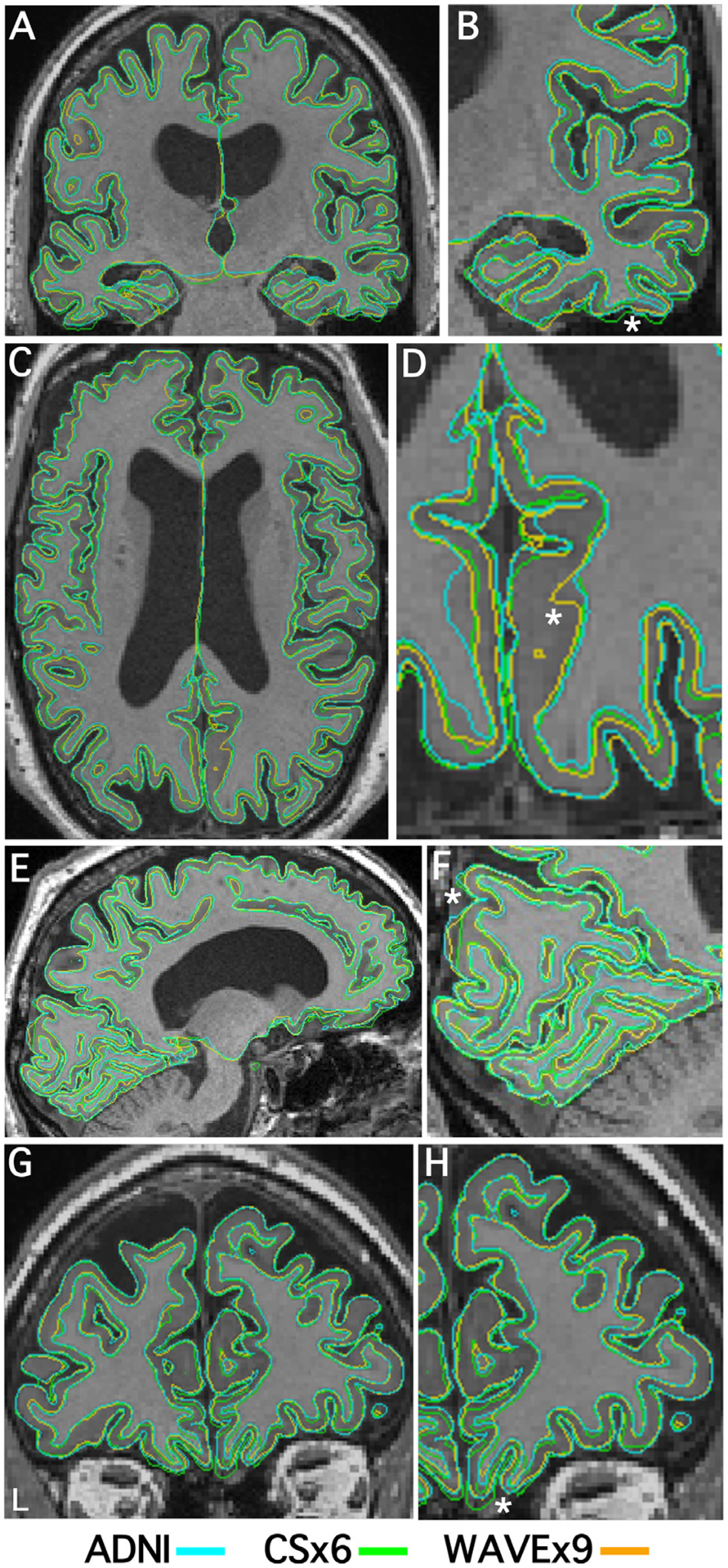
Cortical surfaces align across much of the cortex for all scan types with exceptions. Representative pial surfaces (outer boundaries) and gray/white surfaces (inner boundaries) from a single representative participant (73-year-old male with Alzheimer’s Dementia) are visualized for each scan type simultaneously on top of the same ADNI image (ADNI = cyan, CSx6 = green, and WAVEx9 = orange). Coronal (A, B, G, H), transverse (C, D), and sagittal (E, F) sections are shown at two levels of zoom to aid visualization. This visualization illustrates the similarity in surface estimates across much of the cortex as well as local regions of departure. Local regions of disagreement are illustrated by unclear, messy boundaries where individual boundaries stand out (examples noted by asterisks). For example, in the orbitofrontal prefrontal cortex (a region with relatively low test-retest reliability across scan types), each scan type has a different surface estimate (H, zoom). Estimation errors of this type contribute to the regions with lower reliability and validity estimates in Fig. 6.

Figs. 1–3 were generated using the freeview tool in FreeSurfer. All other figures were generated using version 3.3.5 of the ggplot2 package within R version 4.1.0.

## 3. Results

### 3.1. Rapid scans produce morphometrics-ready images

In a sample of older adults that included individuals with neurodegenerative dementia (Table 1), both WAVEx9 and CSx6 scans produced high-quality T1-weighted images and morphometric measures in approximately 1/5th of the acquisition time as the widely used ADNI scan. This result is the core finding of this paper that is supported by multiple analyses.

Fig. 1 displays coronal and sagittal sections in a representative participant from the ADNI scan (acquisition time = 5′12”) as well as equivalent slices from the CSx6 (acquisition time = 1′12”) and WAVEx9 scans (acquisition time = 1′09”). ADNI scans generated crisp images with clear gray matter-white matter boundaries. These qualities are reflected in image quality metrics, including high signal-to-noise ratio, contrast-to-noise ratio, and relatively low spatial smoothing (Table 2). Both ADNI and rapid scans produced visually similar images (Fig. 1), but the rapid scans had slightly lower signal-to-noise ratios in white matter and gray matter (Table 2). Notably, the contrast-to-noise ratio, which reflects the separability of gray and white matter, was largely unaffected by CSx6 or WAVEx9 acceleration. In addition, while the spatial smoothness of CSx6 images was minimally affected by acceleration, the images based on WAVEx9 acceleration (as reconstructed with our chosen parameters) resulted in additional spatial smoothing that is apparent in Fig. 1 and quantified in Table 2.

Fig. 2 illustrates the consistency of subcortical labeling across scan types in a representative cognitively unimpaired participant. Fig. 3 illustrates the general convergence of white matter and gray matter surface generation in an older adult with Alzheimer’s dementia. Despite the reduction in acquisition time and the differences in image quality metrics between scans, to the eye, automated segmentation and labeling appeared to perform consistently in rapid scans and ADNI. The remaining analyses quantify the performance of the rapid scan variants in comparison to the ADNI scan.

### 3.2. Morphometric measures from rapid scans are highly reliable

High test-retest reliability of morphometric measures is necessary for the investigation of between-subject differences and within-subject longitudinal change. Therefore, if rapid T1-weighted acquisitions are to be useful, they must have high test-retest reliability that performs similarly to standard alternatives like ADNI.

**Fig. 4.**
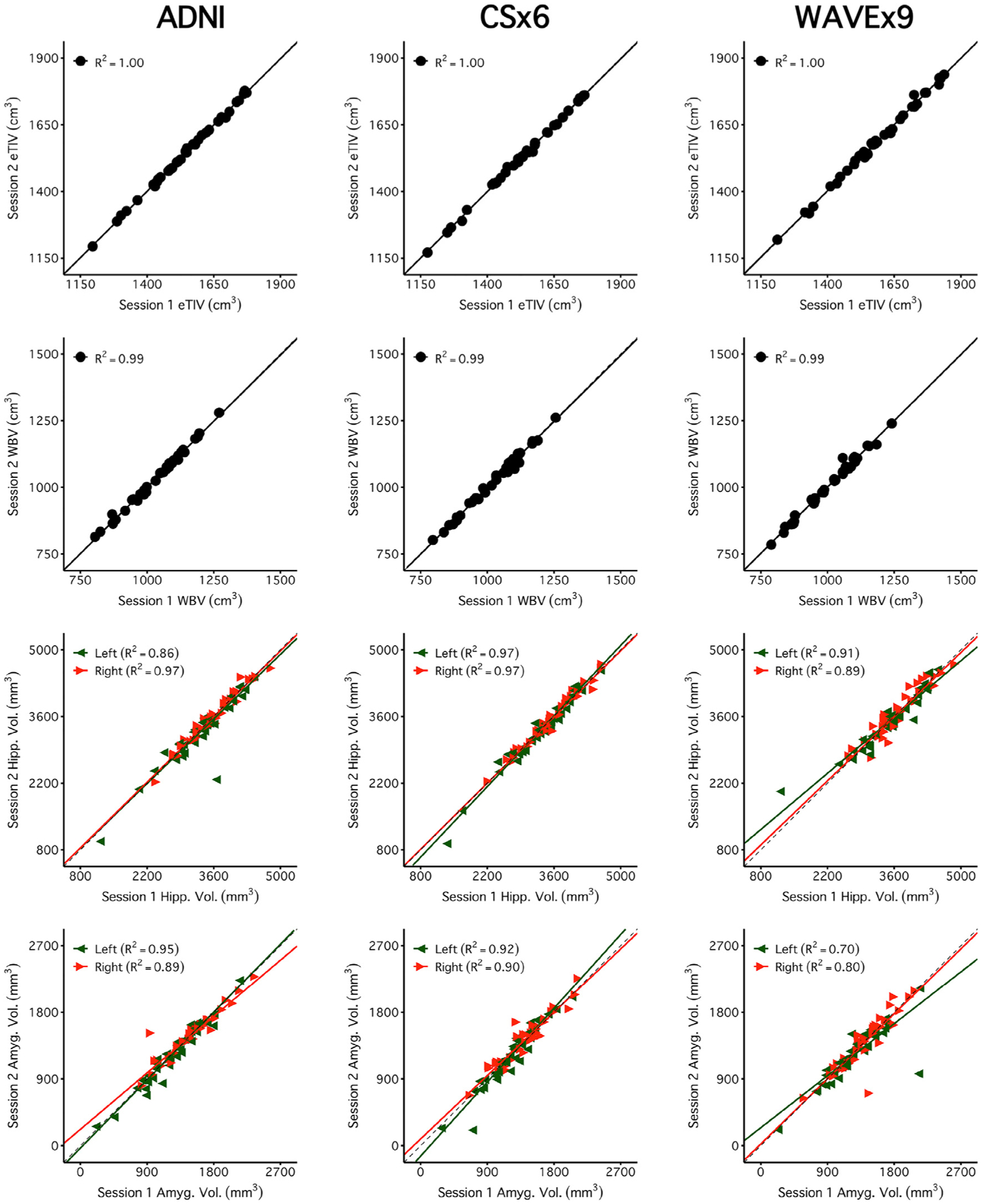
Brain volume measures are highly reliable across days including measures from rapid structural sequences. Each plot displays the test-retest reliability brain volume measures that were independently estimated from two scan sessions on separate days. The between-subject correlation between volumetric measures from session 1 (x-axis) and session 2 (y-axis) are displayed for each scan type (columns) and four separate brain volume measures (rows). The four morphometric measures were selected to possess varied reliability from highest (top) to lowest (bottom). The size of each test-retest correlation (R^2^) is displayed in the top left of each panel. The first two rows display two widely used global brain volume measures – estimated total intracranial volume (eTIV) and whole brain volume (WBV). The third and fourth rows display measures of hippocampal volume (Hipp. Vol.) and amygdala volume (Amyg. Vol.). For these bilateral regional volume measures, estimates from each hemisphere are plotted separately (green triangles for the left and red triangles for the right). Perfect agreement (*X* = Y) is displayed in each plot as a dotted identity line. Generally, these plots illustrate excellent test-retest reliability, even displaying reliable estimation for the cases of neurodegeneration (the lowest values in the hippocampal and amygdala plots). Note that while ADNI and CSx6 reliabilities are similar in each case, the reliability for ADNI hippocampal volume and WAVEx9 amygdala volume are lower due to outlier measures in an individual with svPPA where the temporal lobe has marked neurodegeneration. While uncommon, these examples highlight how outliers occur in both ADNI and rapid scans.

**Fig. 5.**
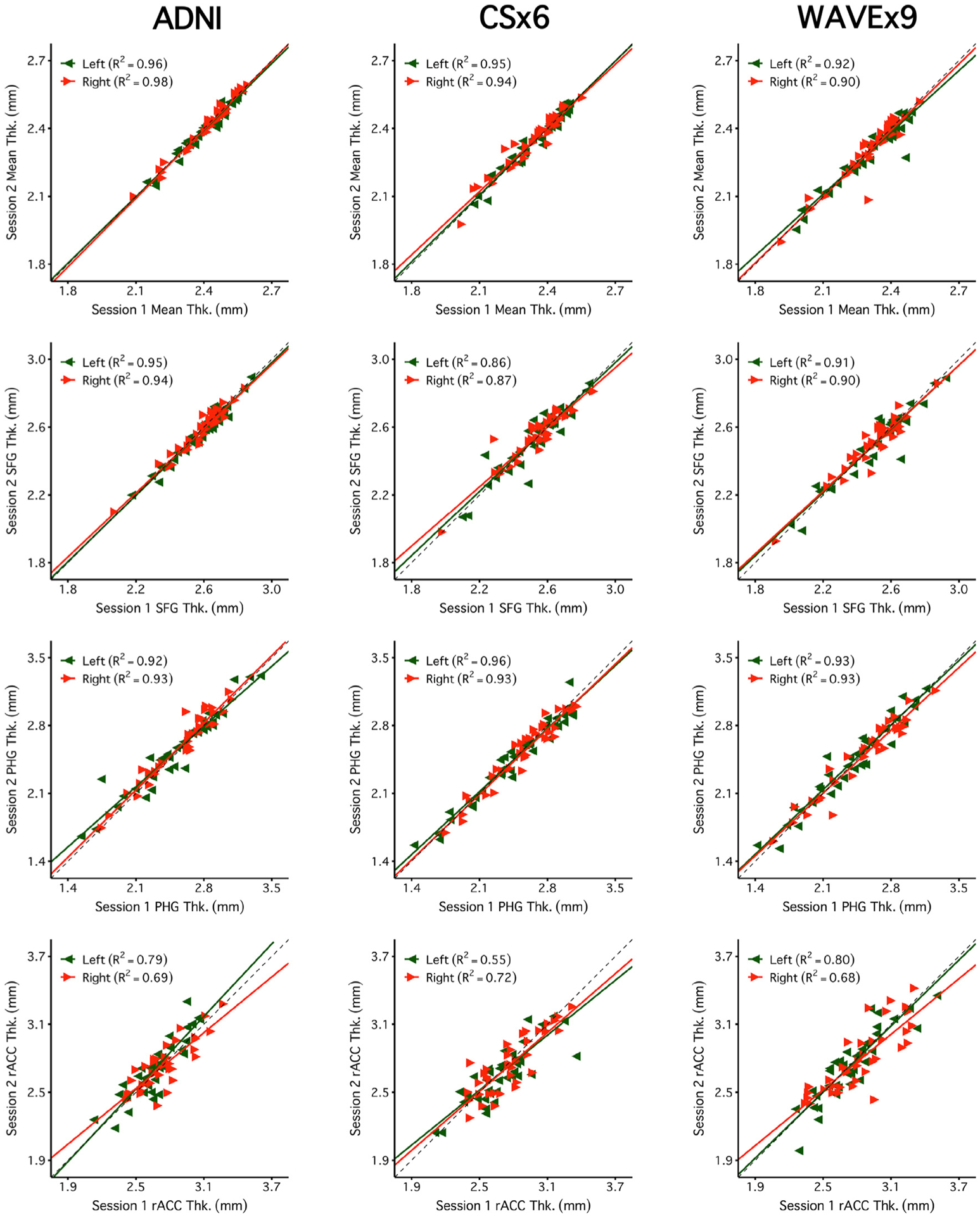
Regional cortical thickness measures are highly reliable across days including measures from rapid structural sequences. Each plot displays the test-retest reliability of regional thickness measures that were independently estimated from two scan sessions on separate days. Plots are arranged as in Fig. 4 with scan types in each column and regional thickness measures in each row. The four example measures were again selected to possess varied reliability from highest (top) to lowest (bottom). The first row is a global measure of mean cortical thickness across the entire cortex (Mean Thk.). Next are rows displaying regional thickness measures including the superior-frontal gyrus (SFG Thk.), the parahippocampal gyrus (PHG Thk.), and the rostral anterior cingulate (rACC Thk.). The rapid scans perform similarly to ADNI. Notably, reliability estimates were lower across all scans for the rACC, a region with known estimation challenges.

Figs. 4 and 5 reveal a consistent pattern of high reliability across scan types for several example measures of interest. Specifically, all scans had excellent reliability in global measures (eTIV, WBV, mean cortical thickness) and high reliability in several regions of particular interest to studies of aging and neurodegeneration including the hippocampus, amygdala, and parahippocampal gyrus. Notably, ADNI, CSx6, and WAVEx9 scans all had moderate reliability in the rostral anterior cingulate, a region where gray-matter and white-matter boundaries are difficult to estimate, even for the reference ADNI scans.

**Fig. 6.**
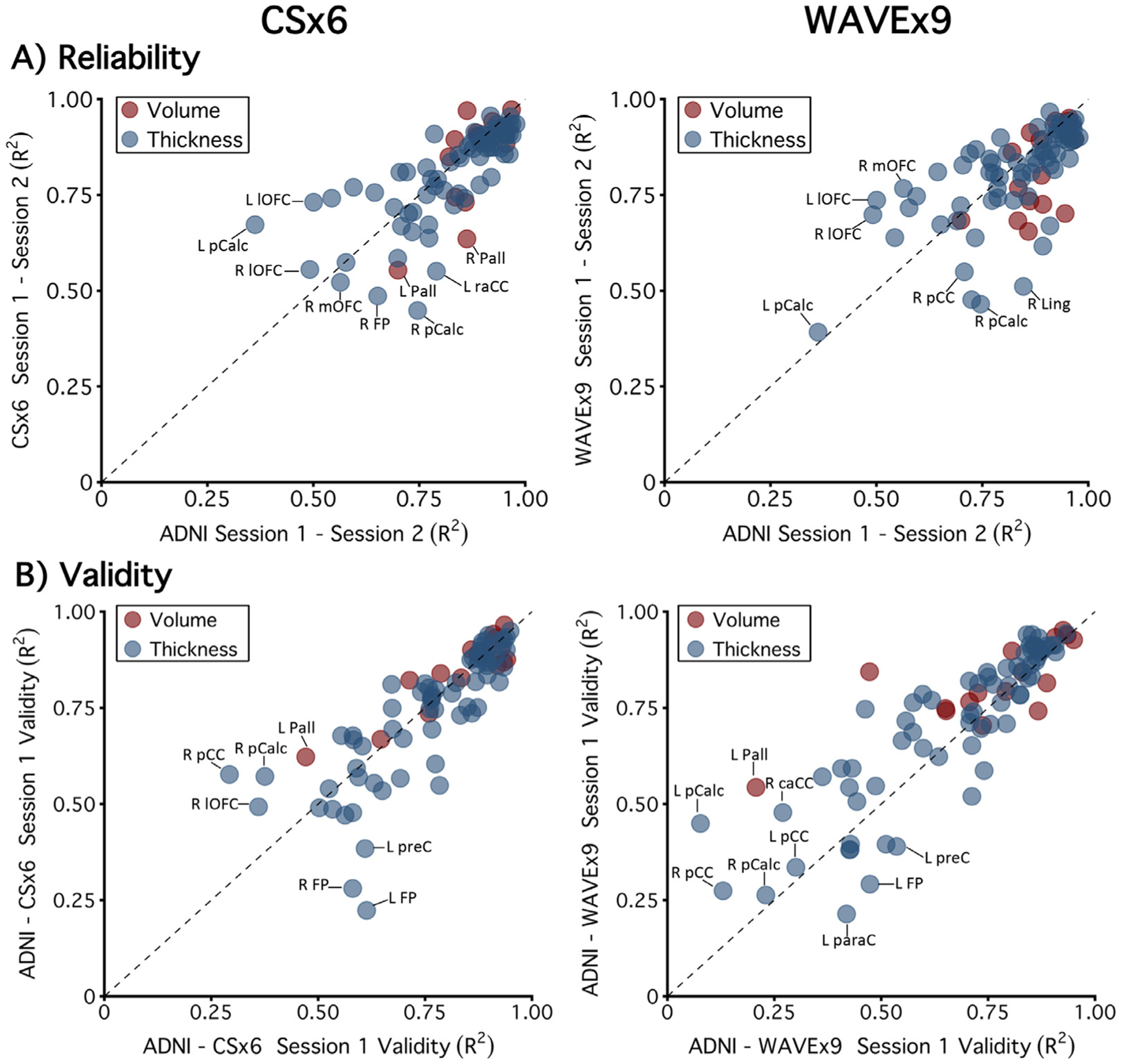
Reliability and validity estimates for all measures. The top row (A) extends from the data presented in Figs. 4 and 5, to compare the test-retest correlations (R^2^) between ADNI (x-axis) directly to each of the rapid scan types (y-axis) for all measures. Correlation values are plotted for CSx6 (left) and WAVEx9 (right). Volume measures are in red and thickness measures are in blue. Most correlations are clustered along the identity line in the upper right-hand corner indicating that estimates are similar between scan types and highly reliable. Several regions in the orbitofrontal cortex are above the identity line indicating that they have higher reliabilities in both CSx6 and WAVEx9 than in ADNI. Conversely, several regions that are located near the midline of the brain (e.g., pallidum and cingulate cortex) are found below the identity line indicating that they have higher reliabilities in ADNI than in both rapid scan types. Notably, these regions are furthest away from the head coil and thus may be most impacted by lower SNR in the rapid scans. The bottom row (B) comprehensively displays the validity estimates for all measures. All regional validity estimates are plotted from correlations (R^2^) between ADNI and each rapid scan type (CSx6 on the left and WAVEx9 on the right). Each plot displays Session 1 validity estimates (x-axis) plotted against Session 2 validity estimates (y-axis). Validity estimates for volume measures are plotted in red and thickness measures are plotted in blue. Most validity estimates are clustered in the upper right of the plot along the *X* = Y identity line indicating strong convergent validity that is consistent across sessions. Regions away from the identity line, including the lateral orbitofrontal cortex, posterior cingulate, and pallidum, highlight regions where validity estimates are inconsistent between sessions and may be affected by outlier estimates in a single session. Abbreviations: left (L), right (R), pallidum (Pall), posterior cingulate cortex (pCC), medial orbitofrontal cortex (mOFC), lateral orbitofrontal cortex (lOFC), caudal anterior cingulate cortex (caCC), rostral anterior cingulate cortex (raCC), peri-calcarine (pCalc), lingual (Ling), frontal pole (FP), precentral (preC), paracentral (paraC).

Fig. 6A directly compares the estimated reliability of each measure across scan types. This analysis serves two purposes. First, it provides a visualization of the reliability of every measure. Second, the plot directly compares reliability across scan types to highlight the relative strengths and weaknesses of the rapid scans. Results revealed that most measures were highly reliable across all three scan types and are clustered in the upper-right corner of Fig. 6A (77% of measures were R^2^ > 0.75 for ADNI, 73% for CSx6, and 69% for WAVEx9). Notably, both CSx6 and WAVEx9 had higher reliability than ADNI in several regions in the orbitofrontal cortex. Conversely, ADNI scans had higher reliability estimates than both CSx6 and WAVEx9 for morphometric measures across several midline brain regions including the cingulate cortex, the pallidum and the pericalcarine cortex. Further investigation revealed that while there was a slight tendency for some smaller regions to have lower reliability, the measures with low reliability were not predictable by size alone (Supplemental Fig. 1).

See the supplementary materials for a complete table of reliability estimates for all morphometric measures.

### 3.3. Measurement errors from rapid scans are comparable to ADNI

**Fig. 7.**
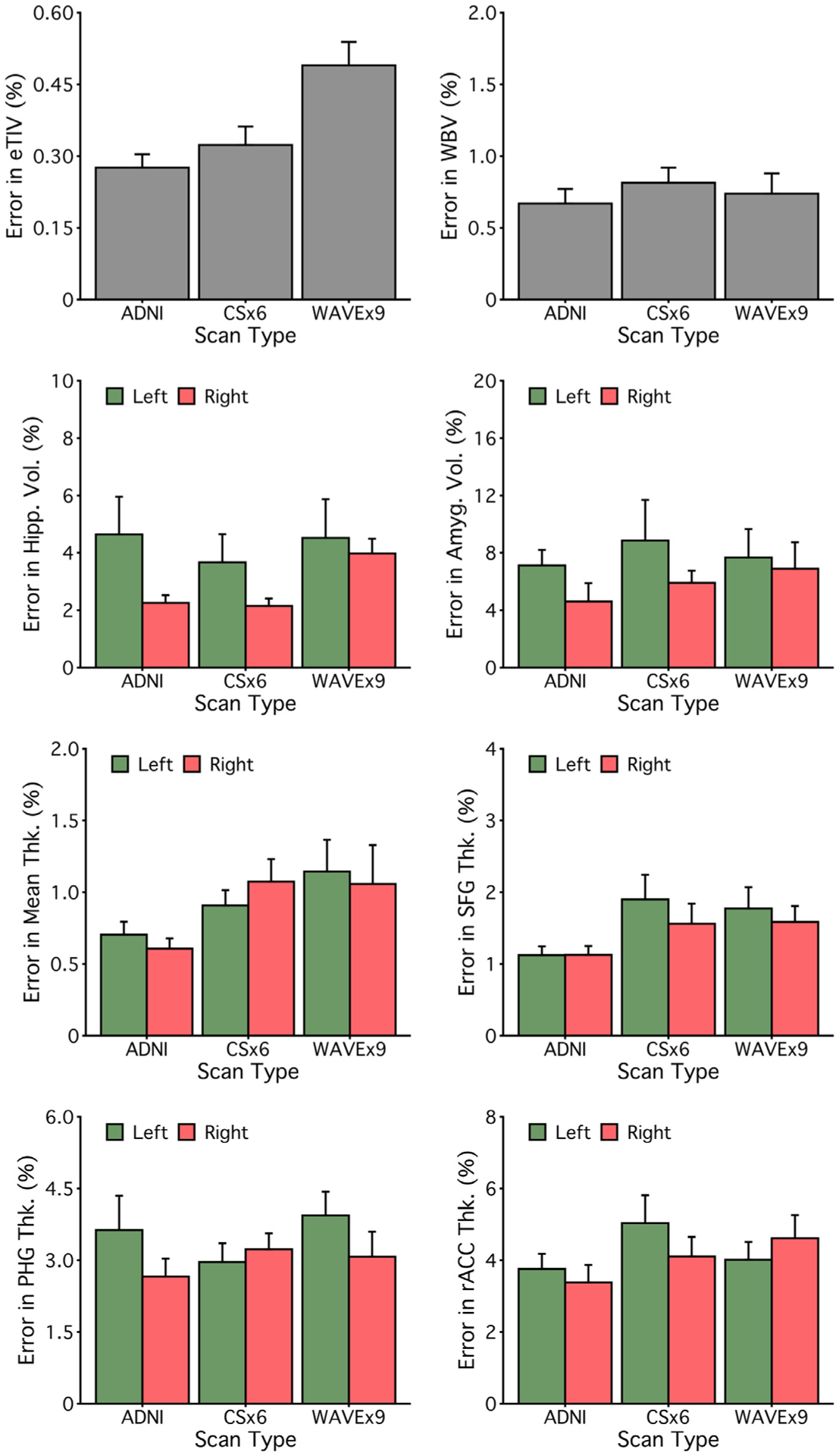
Measurement error is similar across scan types. Mean measurement errors were estimated for each measure from Figs. 4 and 5. For each morphometric measure, the measurement error is estimated as percent errors, defined as the absolute difference between the Session 1 and Session 2 estimates divided by the average morphometric size, averaged across all participants. Error bars represent the standard error of the mean. Error estimates are roughly similar across scan types. These results suggest that rapid scans can match ADNI’s precision across many measures of interest, including widely used regional measures like hippocampal volume.

Measurement error limits statistical power when estimating differences between groups or longitudinal change within an individual (and therefore within a group). We estimated the percent measurement error for each morphometric measure as the average absolute difference between the estimated measure from Session 1 and Session 2 divided by the average size of each morphometric measure. Measurement error varied across morphometric measures of interest with similar measurement errors across scan types (Fig. 7).

**Fig. 8.**
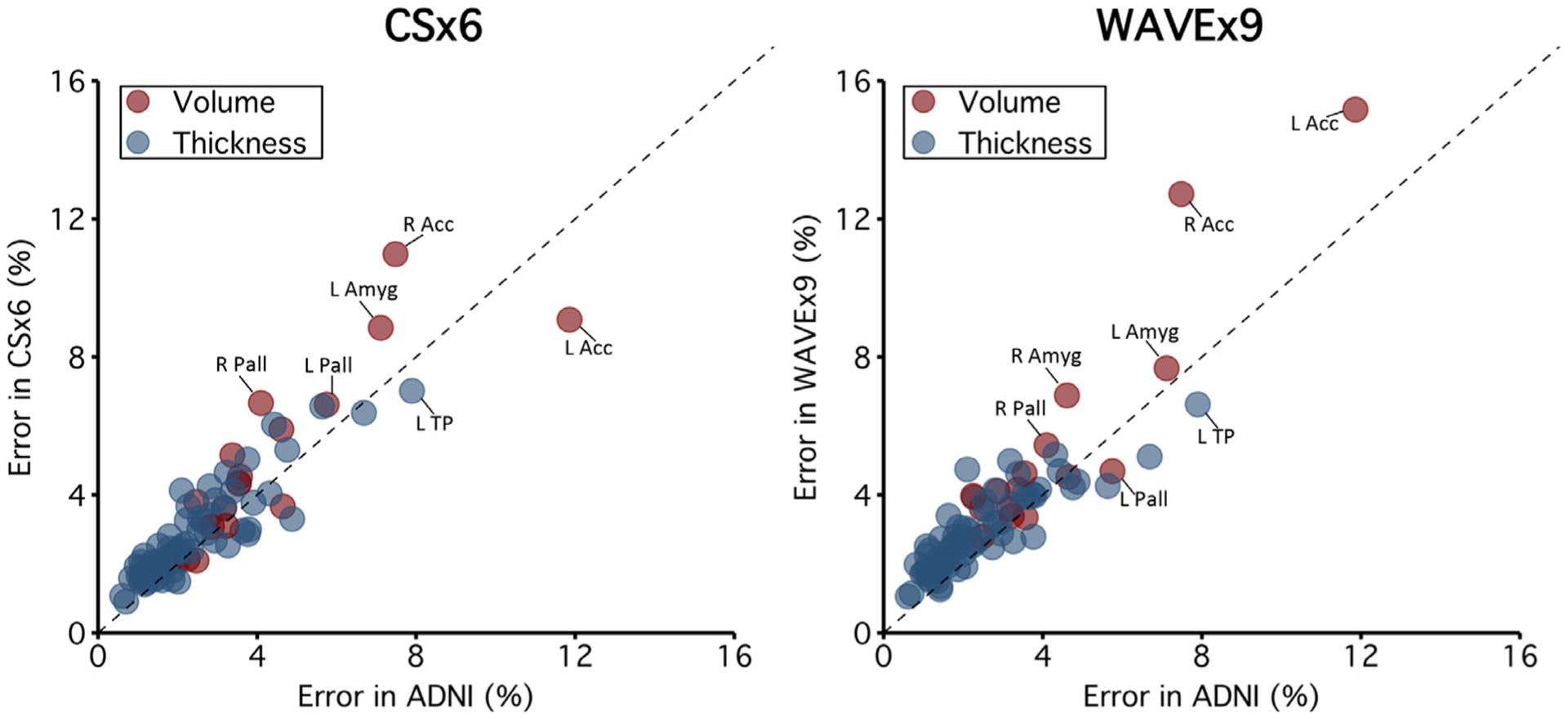
Measurement error estimates for all regions. Extending from the data presented in Fig. 7, which illustrates measurement error estimates for individual measures, the present plots comprehensively show the error estimates for all measures. Error estimates are plotted for CSx6 (left) and WAVEx9 (right) against error estimates for ADNI. Errors for volumes are plotted in red and thickness in blue. In most cases, error estimates fall near the *X* = Y identity line indicating similar regional errors in both rapid and ADNI scans. In aggregate, more estimates fall above the identity line than below indicating that, while the differences tend to be small, the rapid scans tend to have larger error estimates. Regions with notably larger errors in rapid scans compared to ADNI include regions along the midline (e.g., the pallidum) (see also Fig. 6). Across scan types, error estimates were the largest in the accumbens which is a small region known to be influenced by susceptibility artifacts. Abbreviations: left (L), right (R), accumbens (Acc), amygdala (Amyg), pallidum (Pall), temporal pole (TP).

Fig. 8 compares measurement errors for all measures across 1.0 mm scan types. Overall, measurement errors were roughly similar across scan types across measures. The mean measurement error for ADNI was 2.81% (SD = 3.58%) and 70% of measures had a measurement error of less than 3%. The mean measurement error for CSx6 was 3.28% (SD = 4.21%), and 63% of measures had a measurement error of less than 3%. The mean measurement error for WAVEx9 was 3.44% (SD = 4.43%), and 61% of measures had a measurement error of less than 3%. Despite being approximately 5 times faster to collect, the measurement precision of CSx6 and WAVEx9 scans roughly matched that of the ADNI scan.

Both percent and absolute errors for all measures are comprehensively provided in the supplementary materials.

### 3.4. Morphometric measures from rapid scans are valid

Convergent validity of the morphometric measures was estimated from the rapid scans by directly comparing them to the ADNI scans. The primary estimate of convergent validity from these models was the proportion of variance in each morphometric measure that was shared between estimates derived from the ADNI scans and the rapid scans. A higher proportion of shared variance indicates that the rapid scans captured the same between-subjects variance as the ADNI scans.

**Fig. 9.**
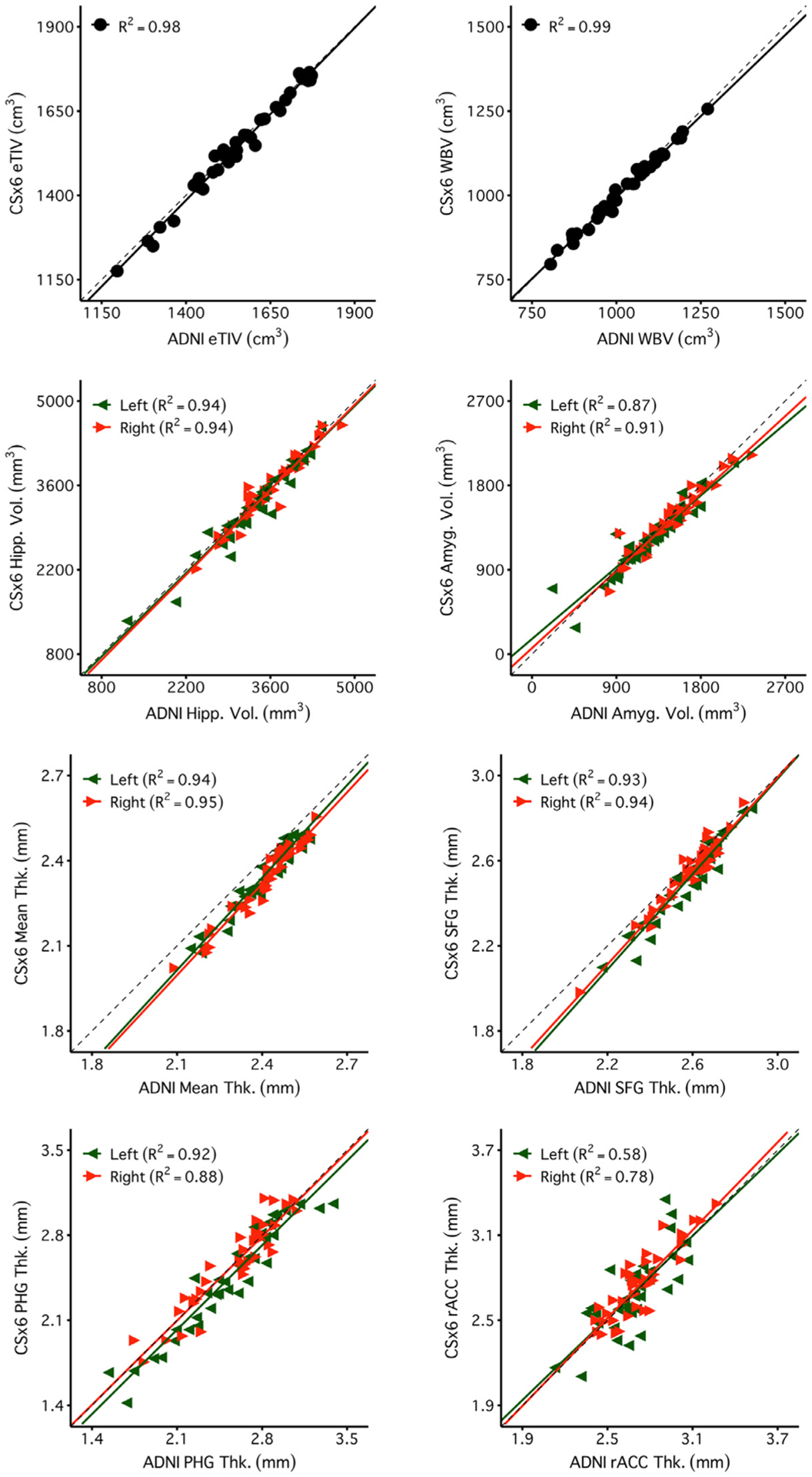
Measures estimated from CSx6 scans are similar to those obtained from the standard ADNI reference scan. Estimates of convergent validity are displayed as the correlation (R^2^) for each measure displayed in Figs. 4 and 5. Plots display the between-subject correlation between brain volume measures estimated from the ADNI images (x-axis) with those estimated from the CSx6 images (CSx6; y-axis). Given each set of scan types was collected over two sessions, two separate R^2^ estimates are available. Session 1 is visualized here, and Session 2 is displayed in Supplemental Figure 2. High correlations are replicable across both sessions and closely cluster along the *X* = Y identity line, indicating a high degree of validity for the extremely rapid CSx6 scans. Note, the values for mean thickness fall off the identity line but remain proportionate across scan types with a high R^2^. This mean shift is likely due to different contrast properties between the ADNI and CSx6 scans leading to a subtle shift in the automated placement of gray/white boundaries that is made clearest in the global measure of mean thickness.

**Fig. 10.**
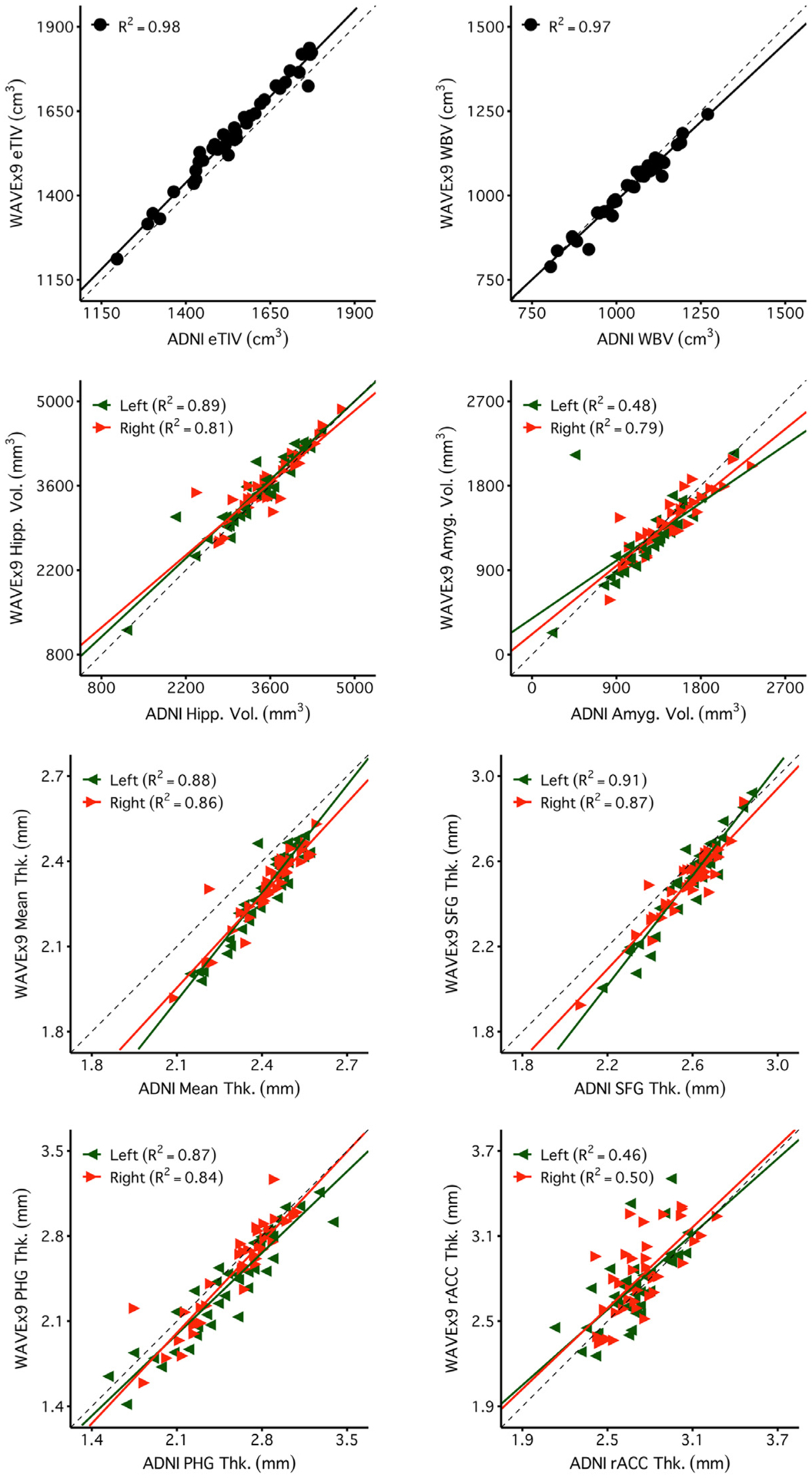
Measures estimated from WAVEx9 scans are similar to those obtained from the standard ADNI reference scan. Estimates of convergent validity are displayed as the correlation (R^2^ ) for each of the regional cortical thickness measures displayed in Figs. 4 and 5. Plots display the between-subjects correlation between the thickness measures estimated from the ADNI images (x-axis) and those estimated from the CSx6 images (CSx6; y-axis). Given each set of scan types was collected over two sessions, two separate R^2^ estimates are available. Session 1 is visualized here, and Session 2 is displayed in Supplemental Figure 3. High correlations are generally replicable across both sessions and closely cluster along the *X* = Y identity line, indicating a high degree of validity for the extremely rapid CSx6 scans. Note, the values for mean thickness fall off the identity line but remain proportionate across scan types with a high R^2^. This mean shift is likely due to different contrast properties between the ADNI and CSx6 scans leading to a subtle shift in the automated placement of gray/white boundaries that is made clearest in the global measure of mean thickness.

Global and regional volumes and cortical thickness measures from the CSx6 scans generally converged with the ADNI scans (Fig. 9). Similar results were found for WAVEx9 vol and cortical thickness measures (Fig. 10). Furthermore, these high convergent validity estimates in CSx6 and WAVEx9 were replicated in independent data from Session 2 (Supplemental Figs. 2 and 3). Across all morphometric measures, both CSx6 (64% of convergent validity estimates were R^2^ > 0.75, 93% R^2^ > 0.50) and WAVEx9 (51% of convergent validity estimates were R^2^ > 0.75, 82% R^2^ > 0.50) demonstrated generally high convergent validity with ADNI that was consistent across sessions (Fig. 6B). Unsurprisingly, convergent validity was higher for morphometric measures that also had high test-retest reliability (e.g., the superior frontal gyrus and the parahippocampal gyrus) and lower for regions including the rostral anterior cingulate (rACC), where reliability was consistently low across scan types (see Figs. 5 and 6A). Additionally, we constructed Bland-Altman plots to further evaluate agreement and look for measurement bias (Supplemental Figs. 4–7). The Bland-Altman plots found widespread agreement between rapid scans and ADNI with minimal measurement bias. Together, these findings suggest that low reliability likely attenuated at least some of the observed convergent validity estimates. Convergent validity estimates for all measures are comprehensively provided in the supplemental materials.

To further investigate the impact of reliability on convergent validity, we used the Spearman correction for attenuation to estimate the theoretic validity of each morphometric measure if they each had been estimated with perfect reliability (Spearman, 1904). After applying the Spearman correction, convergent validity estimates improved substantially for CSx6 morphometric measures (94% of adjusted convergent validity estimates were R^2^ > 0.75, 98% R^2^ > 0.50) and WAVEx9 (78% of adjusted convergent validity estimates were R^2^ > 0.75, 92% R^2^ > 0.50), indicating that lower validity estimates were largely driven by measurement unreliability and marginally due to other underlying differences in measurements between rapid scans and the ADNI scans.

Next, we estimated a proxy for the sensitivity of the CSx6 and WAVEx9 scan estimates by extracting the slope from the convergent validity linear regression models. Morphometric measures estimated from rapid scans could be reliable and highly correlated with estimated measures from ADNI scans while still having less sensitivity to detect variability across participants. While longitudinal data were not available, the relative sensitivity of each scan type to between-subject variance can be used as a proxy for sensitivity to detect longitudinal change. If a 1 unit change in the ADNI morphometric measure yields significantly less than a 1 unit change in the rapid scan measure, this would suggest lower sensitivity even if the error and reliability estimates were similar. We found that, on average, there was minimal loss of sensitivity compared to ADNI scans for both CSx6 (70% of slopes were > 0.90) and WAVEx9 morphometric scans (66% of slopes were > 0.90). In Figs. 9 and 10, reduced sensitivity is illustrated by a shallower line of best fit whereas potentially enhanced sensitivity is illustrated by a steeper line of best fit when compared to the unit line.

### 3.5. Reliability and validity outliers suggest opportunities for rapid scan improvement

Brain regions vary widely in shape, size, and susceptibility to artifacts (due to their positioning relative to the head coil, sinuses, and ventricles). Rapid scans may be differentially affected by local artifacts due to variations in their k-space sampling trajectories. This would generate measurement challenges for morphometric measures in individual brain regions but may be masked by the aggregate summaries of reliability and validity estimates presented above. To investigate this possibility, we searched for regional outliers in several diagnostic comparisons of rapid scans with ADNI (Figs. 6 and 8 and Supplemental Fig. 1).

As expected, small brain regions tended to have morphometric measures with lower test-retest reliability, however, there were notable exceptions (Supplemental Fig. 1). Morphometric measures in the lateral and medial orbitofrontal cortices had low reliability in both rapid and ADNI scans (Fig. 6A) and, consequently, low convergent validity (Fig. 6B), even though the lateral and medial orbitofrontal cortices are about average in size. Poor reliability in these instances is likely due to inconsistent gray matter and white matter boundary detection caused by susceptibility artifacts from paranasal sinuses near the orbitofrontal cortex (Fig. 3). In pilot studies of the CS scans, we used a sagittal acquisition orientation and found even more severe susceptibility-induced artifacts in the orbitofrontal cortex. We found that coronal acquisition orientation mitigated this artifact which led us to adopt a coronal acquisition for this study.

Conversely, several regions of the cingulate cortex had morphometric measures that were outliers in Figs. 6 and 8 because they had moderate to high ADNI test-retest reliability alongside relatively low convergent validity. These brain regions tended to have morphometric measures with higher reliability in the ADNI scan than in CSx6 or WAVEx9 scans (e.g., R^2^ reliability in the right posterior cingulate cortex was 0.71 in ADNI and 0.67 in CSx6 compared to 0.55 in WAVEx9). Similarly, several regions of the medial occipital cortex had morphometric measures from the CSx6 and WAVEx9 scans that were outliers in Fig. 6. These morphometric measures included the thickness of the pericalcarine cortex and lingual gyrus, which appear driven by low test-retest reliability in rapid scans despite moderate to high reliability in the ADNI scans. Notably, these midline regions are furthest away from the head coil and thus may be most impacted by lower SNR in the rapid scans. These outliers highlight the relative regional strengths and weaknesses of each scan type as well as opportunities for further development.

### 3.6. Sub-Millimeter and sub-minute rapid scans are feasible

**Fig. 11.**
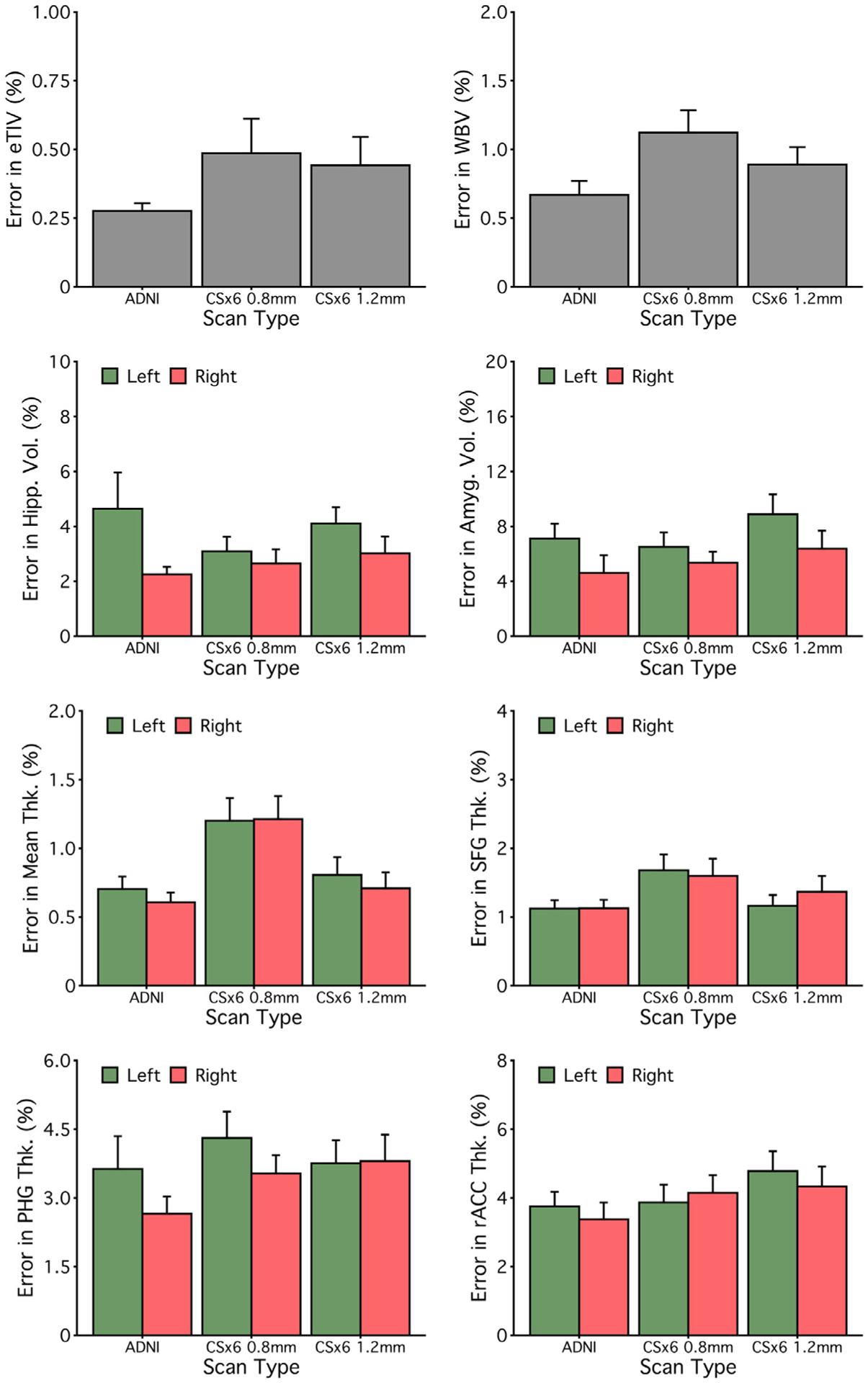
Extremely rapid scans of under one minute may be viable. Measurement errors were estimated for two new scan types that differed in resolution: CSx6 at 0.8 mm (1′49″, matching the resolution used by the Human Connectome Project in Aging; Bookheimer et al., 2019) and CSx6 at 1.2 mm resolution (0′49″, matching the resolution used by the Brain Genomics Superstruct Project; Holmes et al., 2015). For each measure, the percent error is defined as the absolute difference between Session 1 and Session 2 estimates divided by the average size of each morphometric, averaged across all participants. Error bars represent the standard error of the mean. Error estimates are roughly similar to ADNI scans for the sub-millimeter (CSx6 0.8 mm) and the sub-minute (CSx6 1.2 mm) scans. Extremely rapid lower-resolution scans may be viable for quantitative morphometry. Abbreviations: estimated total intracranial volume (eTIV), whole-brain volume (WBV), hippocampus (Hipp), amygdala (Amyg), mean cortical thickness (Mean Thk.), superior-frontal gyrus thickness (SFG Thk.), parahippocampal gyrus thickness (PHG Thk.), rostral anterior cingulate thickness (rACC Thk.).

The results presented to this point suggest that rapid scans can provide reliable and valid morphometric measures with roughly similar precision as the ADNI scan. We next performed a provisional analysis to explore the limits of accelerated scanning. First, we tested whether measurement error could be reduced with a slightly longer, but higher resolution “sub-millimeter” scan (Table 3). Specifically, we estimated measurement error for morphometric measures from a 1′49” 0.8 mm isotropic CSx6 variant (paralleling the resolution used in the HCP-A; Bookheimer et al., 2019). Despite the higher resolution and longer acquisition time, the sub-millimeter scan did not significantly alter measurement error across morphometric measures of interest (Fig. 11). The mean measurement errors for measures from the CSx6 0.8 mm scan were, on average, similar to the measures derived from the 1.0 mm isotropic ADNI and CSx6 scans despite the higher resolution acquisition (*M* = 3.22%, SD = 3.59%, 62% of measures had a measurement error of less than 3%).

**Fig. 12.**
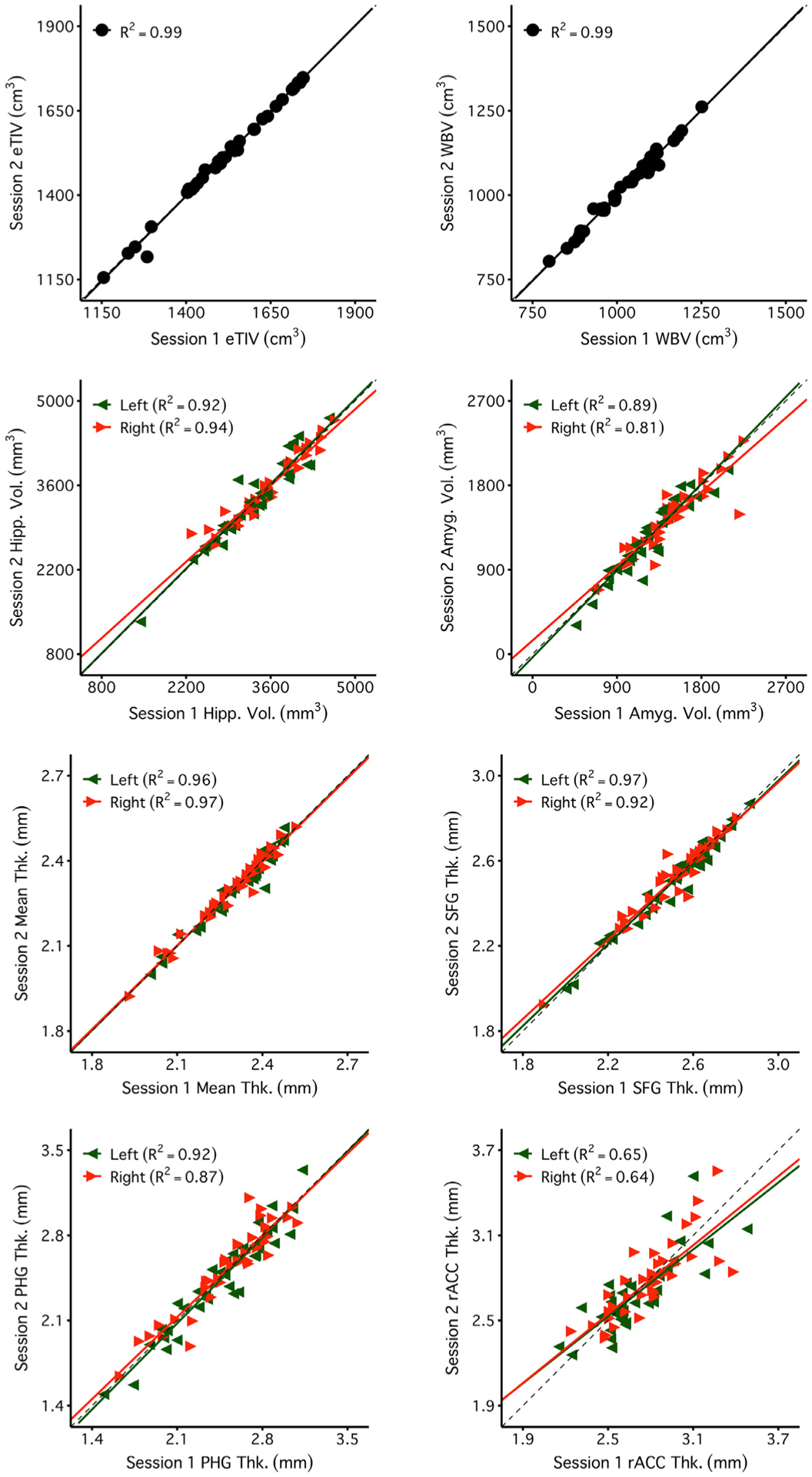
Extremely rapid scans yield reliable measures. Plots display the test-retest reliability estimates for each measure of interest. For each measure, the extremely rapid scan produces highly reliable measures. Despite the larger voxel size, which enables sub-minute scanning, the CSx6 1.2 mm scan performed similarly to the CSx6 1.0 mm scan and the ADNI scan even in small regions like the amygdala. Notably, as in the other scans, reliability estimates were lower for the rACC, a region with known estimation challenges. Abbreviations: estimated total intracranial volume (eTIV), whole-brain volume (WBV), hippocampus (Hipp), amygdala (Amyg), mean cortical thickness (Mean Thk.), superior-frontal gyrus thickness (SFG Thk.), parahippocampal gyrus thickness (PHG Thk.), rostral anterior cingulate thickness (rACC Thk.).

**Fig. 13.**
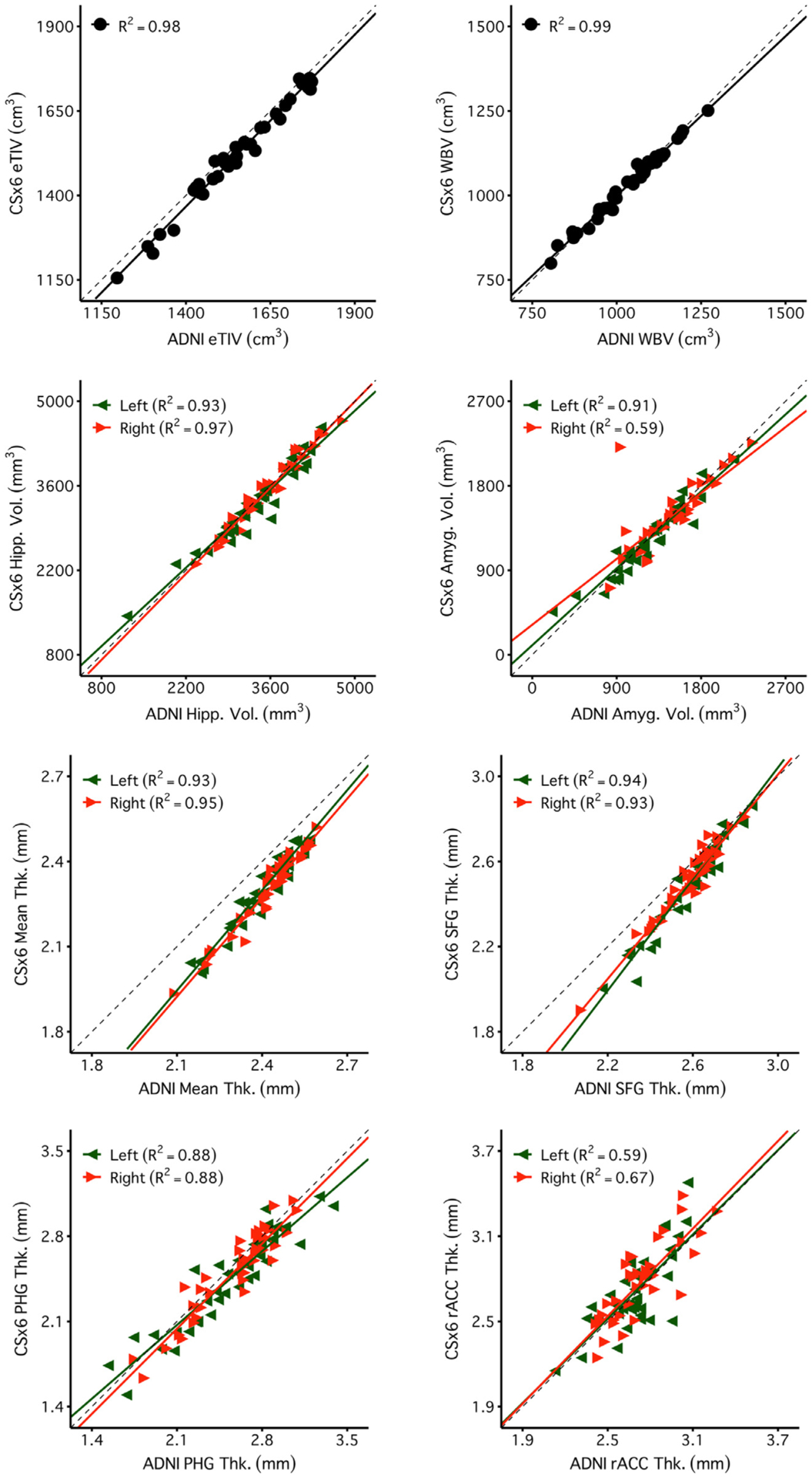
Measures estimated from extremely rapid scans are similar to those obtained from the standard ADNI reference scan. Plots display the convergent validity estimates for each measure of interest. The eight example measures were again selected to possess varied reliability. The extremely rapid scans produce morphometric measures that tend to have high convergent validity despite their larger voxel size and sub-minute acquisition. Note, the values for mean thickness fall off the identity line but remain proportionate across scan types with a high R^2^ . This mean shift is likely due to different contrast properties between the ADNI and CSx6 scans leading to a subtle shift in the automated placement of gray/white boundaries that is made clearest in the global measure of mean thickness. Abbreviations: estimated total intracranial volume (eTIV), whole-brain volume (WBV), hippocampus (Hipp), amygdala (Amyg), mean cortical thickness (Mean Thk.), superior-frontal gyrus thickness (SFG Thk.), parahippocampal gyrus thickness (PHG Thk.), rostral anterior cingulate thickness (rACC Thk.).

Next, we estimated measurement error in a 0′49” 1.2 mm isotropic CSx6 scan variant to test whether even faster scans could achieve comparable morphometric quality (Table 3). The sub-minute 1.2 mm CSx6 scan had similar estimates of measurement precision across morphometric measures of interest (Fig. 11). The mean measurement errors from the CSx6 1.2 mm were similar to those estimated from ADNI and longer CSx6 acquisitions (*M* = 3.31%, SD = 3.69%, 62% of measures had a measurement error of less than 3%). Despite this scan being ~30% faster than even the 1.0 mm CSx6 scan, measurement error was largely unchanged, roughly matching the precision of the 5′12” ADNI scan. Furthermore, the 0′49” CSx6 1.2 mm scan produced reliable morphometric measures with generally high convergent validity (Figs. 12 and 13). As seen in all other scan types, reliability and validity were lower for the rACC. Overall, these results suggest that a sub-minute rapid scan can provide reliable morphometric measures that are similar to measures from standard long scans.

Reliability, error, and validity estimates for the sub-millimeter and sub-minute scans are comprehensively provided in the supplemental materials.

## 4. Discussion

Brain morphometric measures derived from extremely rapid structural MRI scans reliably captured individual differences in a heterogenous sample of older adults with and without dementia. Across an extensive set of quantitative measures, the rapid structural scans provided good performance in 1/5th of the time of a traditional scan. This was true for structural scans based on both compressed sensing and Wave-CAIPI acceleration. In an extreme test, a reduced resolution (1.2 mm) CS scan shorter than one minute in length performed comparably to that of the widely used ADNI scan. We discuss these findings, their implications, and their limitations.

### 4.1. Rapid scans are viable for estimating brain morphometry

Rapid scans represent a significant opportunity for morphometric studies because they offer a practical method to reduce scan session duration and costs, lessen participant burden, and create flexibility for novel biomarker development and research design. ADNI’s structural MRI (acquisition time = 5′12″) was used as our reference to estimate morphometric measures ranging from eTIV and WBV to regional volumes and cortical thickness. With few exceptions, we found that both 1.0 mm CS (1′12″) and Wave (1′09″) rapid scans produced morphometric measures that were highly correlated with ADNI measures and had comparable sensitivity to detect individual differences in a heterogenous older adult sample. The sample included cognitively unimpaired individuals as well as individuals with distinct forms of neurodegenerative dementia. These results suggest that, for many applications, a scan of approximately one minute in length is a viable replacement for longer, standard alternatives. We were surprised by this finding and therefore performed extensive analyses to confirm it.

In an additional set of analyses, we found that using acquisition resolutions slightly above or below the standard 1.0 mm resolution had minimal impact on morphometric estimation (at least within the set of morphometric measures estimated here). Specifically, we explored CSx6 variants with 0.8 mm isotropic resolution (voxel volume = 0.51 mm^3^) and 1.2 mm isotropic resolution (voxel volume = 1.73 mm^3^). Despite a greater than 3-fold difference in resolution between these alternatives, the reliability, precision, and validity of the morphometric measures were similar and comparable to the standard 1.0 mm resolution acquisitions. The acquisition time of the 1.2 mm CSx6 scan variant was 49 s.

In morphometric studies, it is a common practice to accept the costs and burden of long acquisitions based on the assumption that high-quality morphometry requires high-resolution scans, with optimal visual clarity and gray matter / white matter contrast. Approximately fifteen years ago, we and our colleagues performed a series of studies in cognitively unimpaired older adults showing that morphometric analyses were reliable across scanner manufacturers, field strengths, and earlier-generation acceleration techniques using multiple versions of MPRAGE sequences that were typically 8–10 min long and were optimized to follow those assumptions (Dickerson et al., 2008; Han et al., 2006; Jovicich et al., 2009; Wonderlick et al., 2009). The results presented here empirically demonstrate that, for commonly sought morphometric measures, extremely rapid scans collected by way of multiple acceleration strategies and with suboptimal scan resolutions are viable alternatives.

### 4.2. Limitations and future considerations

Our study has several limitations. Compressed sensing and Wave-CAIPI are emerging acceleration strategies with room for further refinement and optimization. In this study, we explored a small subset of the much broader parameter space of CS and Wave scan variants. We chose scan parameters for quantitative morphometry based on established, successful implementations of CSx6 and WAVEx9 (Mussard et al., 2020; Polak et al., 2018) as well as pilot testing in our center. This limitation restricts our ability to establish optimal scan parameters for CS and Wave. However, we found evidence that these rapid scans can produce high-quality morphometric measures across a range of parameters, bolstering the core finding of this paper: rapid scan variants are viable for morphometry despite differences in image quality with standard, longer scans and differences in the exact acquisition parameters used (at least among the parameters tested here).

Second, automated FreeSurfer morphometry performed similarly in CSx6 and WAVEx9 despite the differences between scan acceleration and reconstruction methods, suggesting multiple paths forward for further improvements and adoption. We did not investigate how these rapid scans would be processed by other morphometric analytic pipelines, which should be explored.

Third, the CSx6 and WAVEx9 acquisitions that are presented here were chosen after extensive piloting (Hanford et al., 2021; Mair et al., 2019, 2020). A current limitation of these rapid scans is that the user must choose both an acceleration and regularization level to balance acquisition speed with the noise produced by acceleration and the smoothing that is introduced by the regularization (Bilgic et al., 2014; Mair et al., 2020). We tested a variety of accelerations and regularization levels and found that CSx6 and WAVEx9 were the fastest accelerations that we could achieve while continuing to have adequate image quality (i.e., SNR, CNR, and spatial smoothness). In our piloting, accelerations past CSx6 and WAVEx9 (e.g., CSx8 or CSx10) led to a worsening of image quality and increases in the severity of banding artifacts with only marginal benefits in scan speed (e.g., CSx8 was only ~15 s faster than CSx6) (Hanford et al., 2021; Mair et al., 2020).

Furthermore, even after piloting, we discovered that our implementation of Wave reconstruction was likely over-regularized while, conversely, the reconstruction of our CS was mildly under-regularized. Evidence for Wave over-regularization was most clear in the FWHM image quality metric in Table 2, where WAVEx9 scans have an average smoothness of 4.28 mm, compared to 3.22 mm in ADNI and 3.08 mm in CSx6. In Figs. 1–3, this over-regularization was subtle but noticeable in white matter, where our WAVEx9 scan had more blurring and less sharp boundaries between white matter and subcortical nuclei than the ADNI and CSx6 images. In contrast, close examination of the CSx6 scans revealed a subtle but consistent pattern of mild banding that was most noticeable in the coronal plane even in still participants.

Despite these signs of suboptimal regularization, there were few discrepancies in the morphometric performance between scan types illustrating the robustness of automated morphometry to at least a mild level of suboptimal regularization (Figs. 6 and 8). We hope that documenting these experiences aid researchers who adopt these rapid scans; however, they also highlight the need for CS and WAVE users to thoroughly pilot rapid scans before implementation as the tradeoffs between image quality, acceleration, and regularization may vary by scanner model and sequence.

Fourth, although we report the performance of these rapid scans in older adults with and without neurodegenerative brain diseases, the results are likely relevant for a wide range of research and patient populations. Explorations in future participant groups might expand to include additional types of neurodegenerative disease, other ages (e.g., children), and individuals with neuropsychiatric illness. Fast scans may be particularly valuable for small children and patients for whom movement and compliance are a challenge.

Fifth, while CSx6 and WAVEx9 scans generally had high levels of agreement with the ADNI scan (see Figs. 6, 9, 10, and 13 and supplemental Figs. 2–7), there were some cases of mean shifts in measures (e.g., mean thickness in Figs. 9, 10 and 13 and supplemental Figs. 5 and 7). This shift indicates that our rapid scans have systematically produced smaller estimates of cortical thickness than the ADNI scan. This shift likely results from differences in contrast properties between scan types that create a subtle, but systematic shift in the positional estimates of the cortical surface (e.g., the white matter boundary and/or pial surface). Differences in contrast properties have been commonly found across different T1 acquisitions and scanners (Fujimoto et al., 2014; van der Kouwe et al., 2008). These differences are unlikely to pose problems for studies that adopt rapid scans from the beginning of a study as scans will be self-referencing within the study. However, in ongoing studies, researchers who consider adopting rapid scans should be cautious and perform harmonization studies to compare measurement properties, and consider adjustment techniques like ComBat, before replacing traditional scans with rapid alternatives (Fortin et al., 2018; Pomponio et al., 2020; Radua et al., 2020). One benefit of rapid scans is their low burden. Thus, it may be possible to add a rapid scan variant to existing, ongoing studies to aggregate data for calibration and to locally explore the utility and viability of rapid scans.

Overall, we found that the reliability, precision, and sensitivity of rapid scans roughly matched estimates from the ADNI scan acquisitions across many morphometric measures. However, it is important to note that, in the aggregate, morphometric measures from the rapid scans tended to have slightly lower reliability and precision than morphometric measures derived from ADNI. Specifically, the morphometric quality of rapid scans suffered most in regions, including the pallidum, cingulate, and pericalcarine cortex that are closest to the midline of the brain. These limitations of morphometric measurements in CS and Wave are consistent with known noise amplification effects that tend to be largest along the midline of the brain in regions that are furthest from the receiving heal coil elements (Sartoretti et al., 2018; Wiggins et al., 2006; Yang et al., 2016).

While reliability and precision tended to slightly favor the ADNI acquisition, we also found evidence that rapid scans had higher reliability and precision for morphometric measures in several regions of the orbitofrontal and anterior temporal cortex. Head motion is more likely to occur in longer scans and these regions have previously been found to be most affected by head motion, possibly due to their proximity to sinuses and corresponding proneness to susceptibility artifacts (Alexander-Bloch et al., 2016; Reuter et al., 2015). While acceleration affords many opportunities, our results highlight regional heterogeneity in morphometric quality between ADNI, CS, and Wave scans. Future research is needed to refine these methods and minimize their weaknesses. In the meantime, in some studies where the morphometric precision of specific regions is paramount, standard MPRAGEs may still be beneficial.

A final limitation is that we studied commonly used morphometric measures of global and regional brain volumes and thickness. These measures are used in numerous studies of brain aging, resilience, and neurodegeneration. However, there are many other uses of T1-weighted images including estimation of the T1/T2 ratio as a proxy for myelination (e.g., Baum et al., 2022; Glasser and Van Essen, 2011; Shafee et al., 2015), examination of small structures in the brainstem (Iglesias et al., 2015), examination of hippocampal subfields (Van Leemput et al., 2009; Wisse et al., 2021) and examination of local abnormalities in the cerebral cortex (Lüsebrink et al., 2013; Noh et al., 2014). These examples, which are not meant as an exhaustive list, are a reminder that different experimental goals may require additional analyses of the performance and quality tradeoffs for rapid scans.

### 4.3. Toward precision morphometric measurement in individuals

A straightforward, initial application for rapid scans is to decrease the overall length of comprehensive scanning sessions. For example, as an evolution of the ADNI initiative (Weiner et al., 2017), SCAN has offered a framework for multiple sites to collect uniform data that can then be aggregated (by the National Alzheimer’s Coordinating Center, NACC). The base protocol (a T1-weighted structural image and a fluid-attenuated inversion recovery image) is about 10 min in length. The extended protocol, based on ADNI-3 sequences, is 30 min or more (Gunter et al., 2017). What if a parallel accelerated protocol was constructed that could be collected in its entirety within 5 min? If achieved, the participant burden would be lower and widespread adoption would be possible because this protocol could easily be an add-on to existing studies. For those able to utilize the protocol fully, excess scanner time would be preserved for local research purposes. A challenging but achievable target for the field should be aspiring to a comprehensive MRI protocol for brain aging and neuropsychiatric disorders that is 5 min or less in total length.

Most neuroimaging studies employ a single structural MRI scan to measure participants’ neuroanatomy. We found both the ADNI and the rapid scans had measurement errors in key regions of interest like the hippocampus that are ~2–5% (Fig. 7). This error approximately matches estimates of the amount of neurodegenerative change that occurs in the hippocampus in Alzheimer’s disease over the course of 1 year (hippocampal atrophy in Alzheimer’s disease is 2–4% greater annually than in typical aging; Barnes et al., 2009; Jack et al., 2015; Pini et al., 2016). Therefore, it would not be possible to determine from a single estimate of annual change whether that change was accelerated (neurodegenerative) or due to random measurement error. However, rapid scans allow for multiple, repeated scans to be collected in the time of a single standard structural image. Since the results here suggest that morphometric measures estimated from a single rapid scan nearly match the precision of a standard long structural scan, it is possible that morphometric measures from multiple sequentially acquired rapid scans within a session may be able to be aggregated to drive down measurement error, yielding higher morphometric precision than an individual estimate can achieve on its own.

Preliminary results in young adults suggest that this “cluster scanning” and aggregation can improve the precision of morphometric measures (Nielsen et al., 2019). However, it remains possible that repeated acquisitions within the same scanning session are sufficiently correlated (and the relevant noise sources are day, time, and head position dependent) that within-session scan repetitions yield diminishing returns when pooled. Future analyses are needed to assess strategies to increase precision by pooling estimates across scans, such as repositioning participants and acquiring multiple scans with different resolutions and scan parameters. While this “cluster scanning” is presently uncommon in morphometric studies, due to the cost and burden of long structural scans, the general principle of repeated sampling to improve precision through aggregation is a core measurement strategy in functional MRI, psychometrics, and beyond and should be explored further with rapid scans (Birn et al., 2013; Gordon et al., 2017; Kuder and Richardson, 1937; Sliwinski, 2008). Additional strategies may also be adopted to further improve the precision of morphometrics from rapid scans. For example, rather than using individual biomarkers like hippocampal volume, composite biomarkers could be developed to aggregate information from multiple morphometrics and artificial intelligence-driven postprocessing could be implemented to improve the image quality of rapid scans (Iglesias et al., 2023; Kang et al., 2021).

Continued improvements in measurement precision through “cluster scanning” and other advancements could have large downstream consequences for biomarker development and clinical translation. For example, a major barrier to discovering treatments for Alzheimer’s disease is that clinical trials are costly because they require years of follow-up and large samples size to detect treatment effects in standard outcome measures (Aisen et al., 2022; van Dyck et al., 2022; Veitch et al., 2019; Zetterberg and Bendlin, 2021). If achieved, a high-precision disease-progression biomarker capable of detecting the rate of neurodegenerative change in individuals across 12 to 18 months, may reduce costs and accelerate clinical trials by improving our sensitivity to detect treatment effects and heterogeneity within neurodegenerative disease trials. Our results provide an initial indication that rapid scans are feasible, opening the door to future research to explore these possibilities.

## 5. Conclusions

We evaluated the reliability, precision, and validity of morphometric measures from two rapid structural imaging techniques (compressed sensing and Wave-CAIPI) by comparing their performance directly to a reference structural scan from the latest ADNI protocol. In a sample of older adults, including those with diverse forms of neurodegenerative dementia, CSx6 and WAVEx9 scans produced reliable and precise morphometric measures that roughly matched the performance of the ADNI scan in 1/5th of the scan length. These results suggest that many studies can reduce participant burden and save costs, with minimal tradeoffs, by replacing well-established, long structural acquisitions with rapid scans. Rapid scans may be especially useful in populations that have difficulty with MRI scan compliance and in those who are prone to head motion, including children, older adults, and individuals with neuropsychiatric illness. Furthermore, rapid scans allow for innovative research designs that may enable even higher precision through repeat or “cluster scanning.”

## Supplementary Material

1

2

3

4

## Figures and Tables

**Table 1:** Participant demographics.

Group	Sample Sizes	Age Mean (range)	Sex (M/F)	CDR (0/0.5/1/2)	CDR SOB Mean (range)	CDR + NACC FTLD (0/0.5/1/2)	CDR + NACC SOB FTLD Mean (Range)
Cognitively Unimpaired	18	73.4 (64 – 86)	6/12	18/0/0/0	0 (0 – 0)	0/0/0/0	0 (0 – 0)
MCI/AD	10	71.2 (55 – 83)	4/6	0/5/5/0	4.25 (0.5 – 7)	0/5/5/0	4.25 (0.5 – 7)
FTLD	9	66.6 (54 – 76)	7/2	2/3/2/2	3.72 (0 – 10)	0/3/4/2	5.73 (0.5 – 14)

*Notes*. Abbreviations: mild cognitive impairment (MCI), Alzheimer’s Dementia (AD), Clinical Dementia Rating (CDR), sum of boxes (SOB), National Alzheimer’s Coordinating Center (NACC), Frontotemporal Lobar Degeneration (FTLD).

**Table 2:** Mean image quality metrics for ADNI, CSx6 1.0 mm, and WAVEx9 1.0 mm scan variants.

Scan Type	SNR WM	SNR GM	FWHM	CNR
ADNI	19.58 (1.89) [19.20 – 19.95]	11.22 (0.94) [11.04 – 11.41]	3.22 (0.17) [3.19 – 3.25]	2.84 (0.43) [2.75 – 2.92]
CSx6	15.79 (1.79) [15.44 – 16.14]	10.16 (0.72) [10.01 – 10.30]	3.08 (0.16) [3.05 – 3.11]	2.65 (0.34) [2.58 – 2.72]
WAVEx9	17.98 (3.32) [17.33 – 18.64]	10.39 (1.08) [10.18 −10.60]	4.28 (0.22) [4.24 – 4.33]	2.48 (0.48) [2.39 – 2.57]

*Notes*. Standard deviations for each metric are in parentheses and 95% confidence intervals are in brackets. Abbreviations: signal-to-noise ratio of white matter (SNR WM), signal-to-noise ratio of gray matter (SNR GM), full-width half maximum (FWHM), contrast-to-noise ratio (CNR).

**Table 3:** Mean image quality metrics for ADNI, CSx6 0.8 mm, and CSx6 1.2 mm scan variants.

Scan Type	SNR WM	SNR GM	FWHM	CNR
ADNI	19.58 (1.89) [19.20 – 19.95]	11.22 (0.94) [11.04 – 11.41]	3.22 (0.17) [3.19 – 3.25]	2.84 (0.43) [2.75 – 2.92]
CSx6 0.8 mm	14.27 (1.73) [13.93 – 14.61]	10.51 (0.77) [10.36 – 10.66]	3.21 (0.22) [3.17 – 3.25]	2.47 (0.32) [2.41 – 2.53]
CSx6 1.2 mm	18.75 (2.51) [18.26 – 19.25]	10.97 (0.81) [10.81 – 11.13]	3.11 (0.13) [3.08 – 3.13]	3.02 (0.42) [2.94 – 3.10]

*Notes*. Standard deviations for each metric are in parentheses and 95% confidence intervals are in brackets. Abbreviations: signal-to-noise ratio of white matter (SNR WM), signal-to-noise ratio of gray matter (SNR GM), full-width half maximum (FWHM), contrast-to-noise ratio (CNR).

## Data Availability

Upon publication, the MRI data that was used in this manuscript will be deposited on openneuro.org and the analysis code will be available on github.com/maxwellelliott/rapidMRImorphometry.
