# Supplementary material for: Brain morphometry in older adults with and without dementia using extremely rapid structural scans
Source: Neuroimage. Author manuscript; Available in PMC 2023 Aug 1. (PMC10330834; doi:10.1016/j.neuroimage.2023.120173)
Supplement: 3 [file NIHMS1910689-supplement-3.pdf]

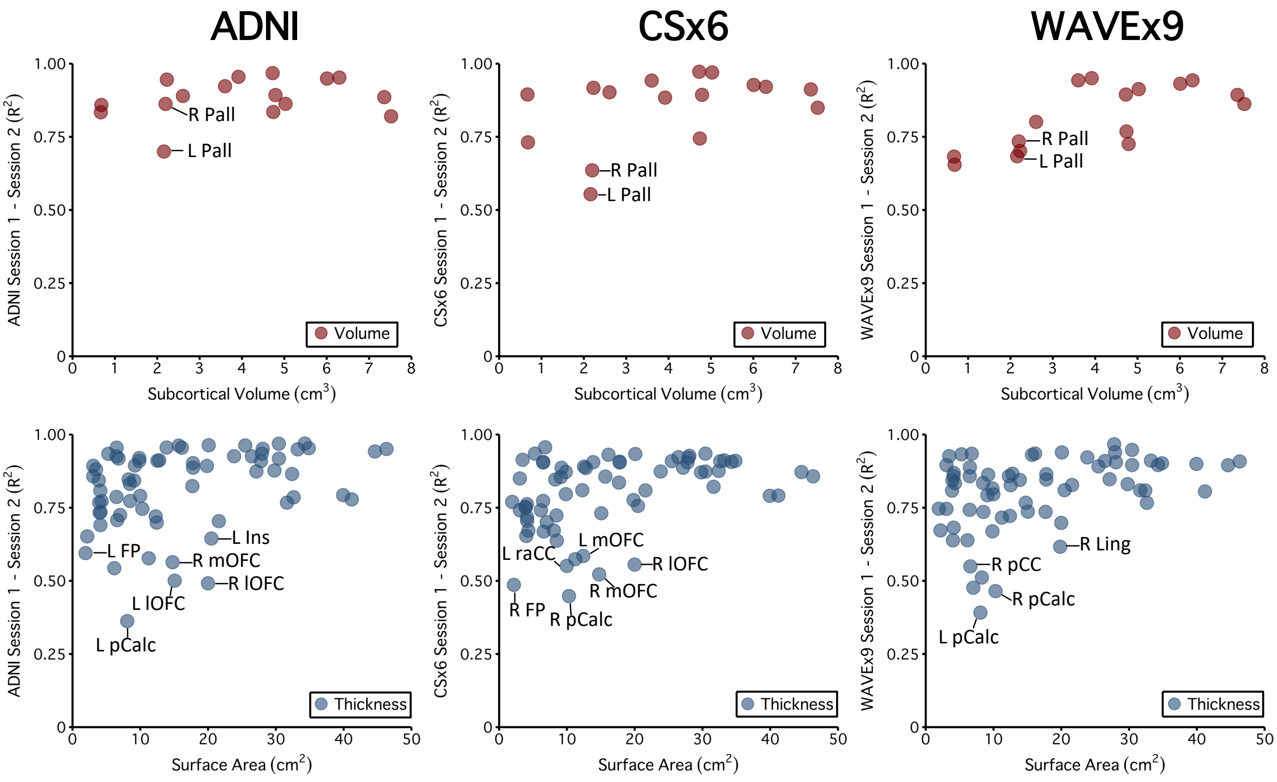

Supplemental Figure 1

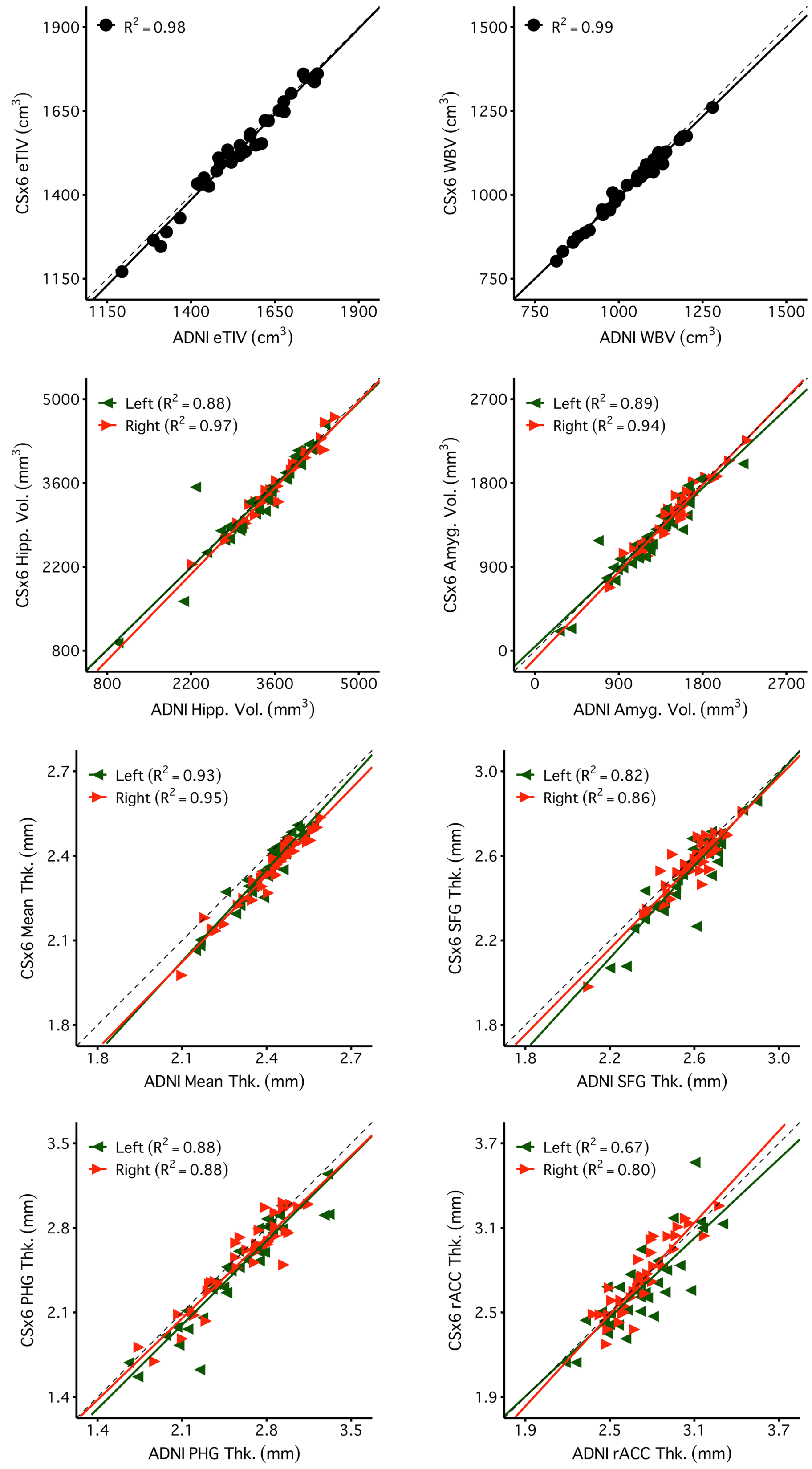

Supplemental Figure 2

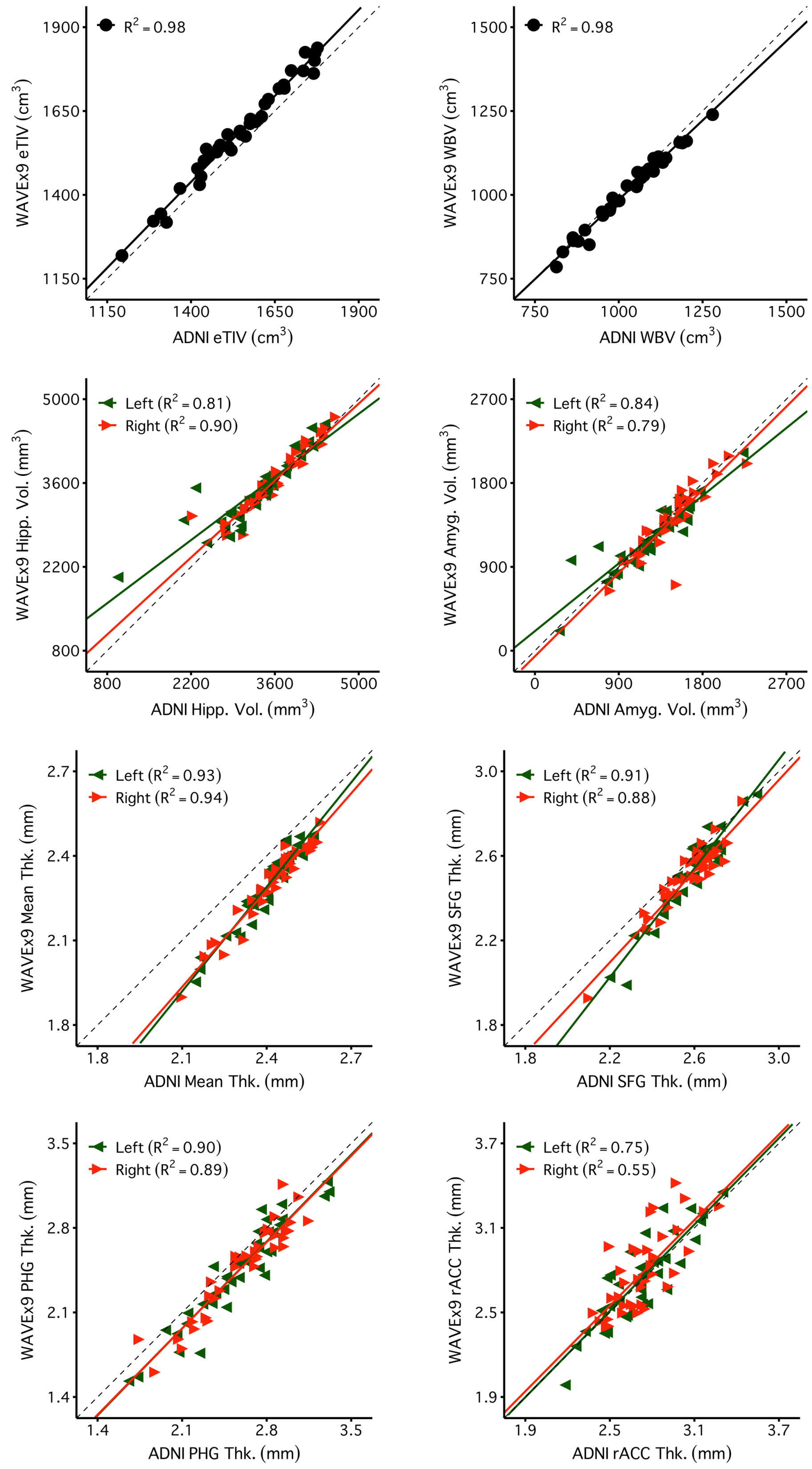

Supplemental Figure 3

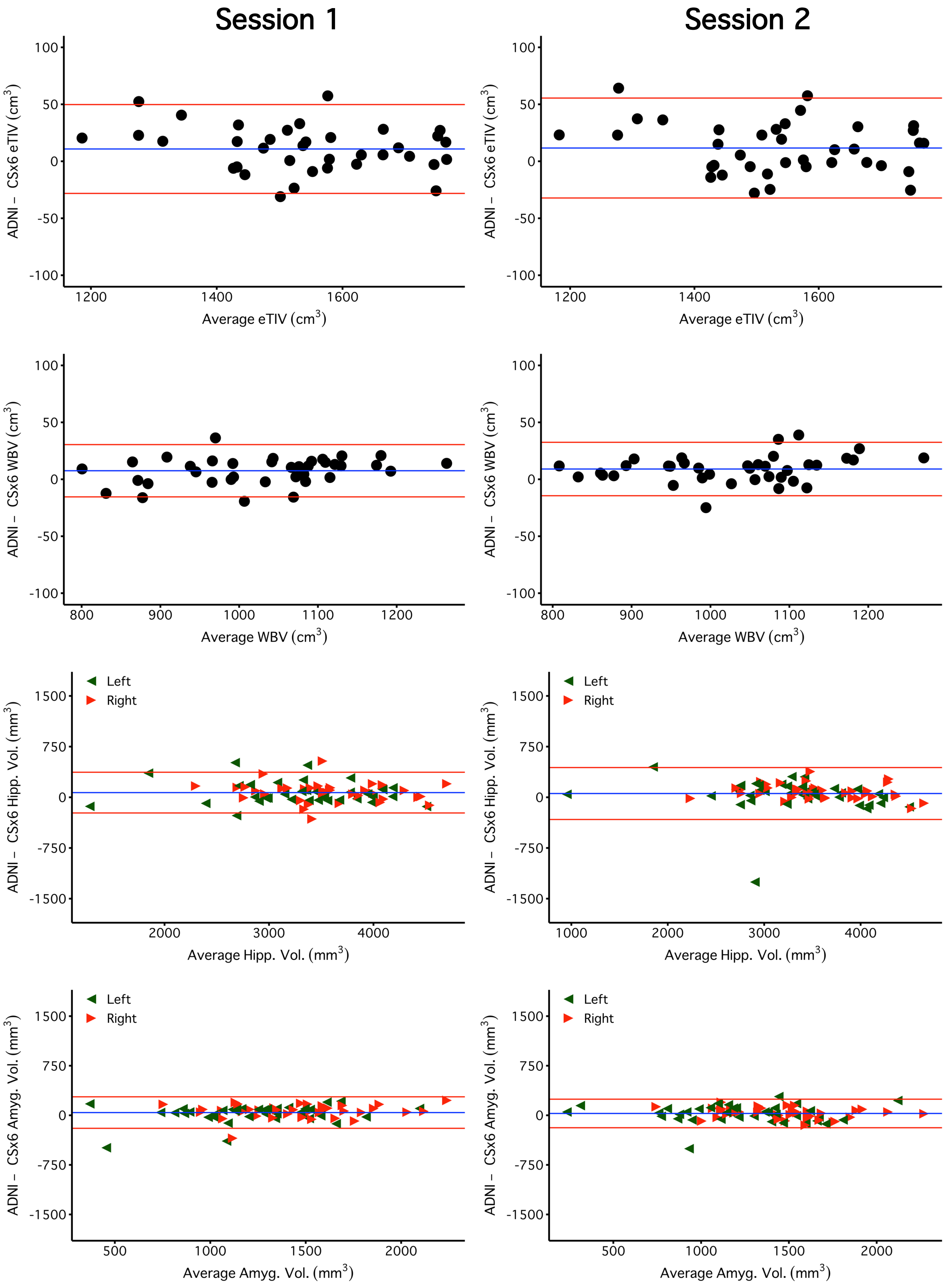

Supplemental Figure 4

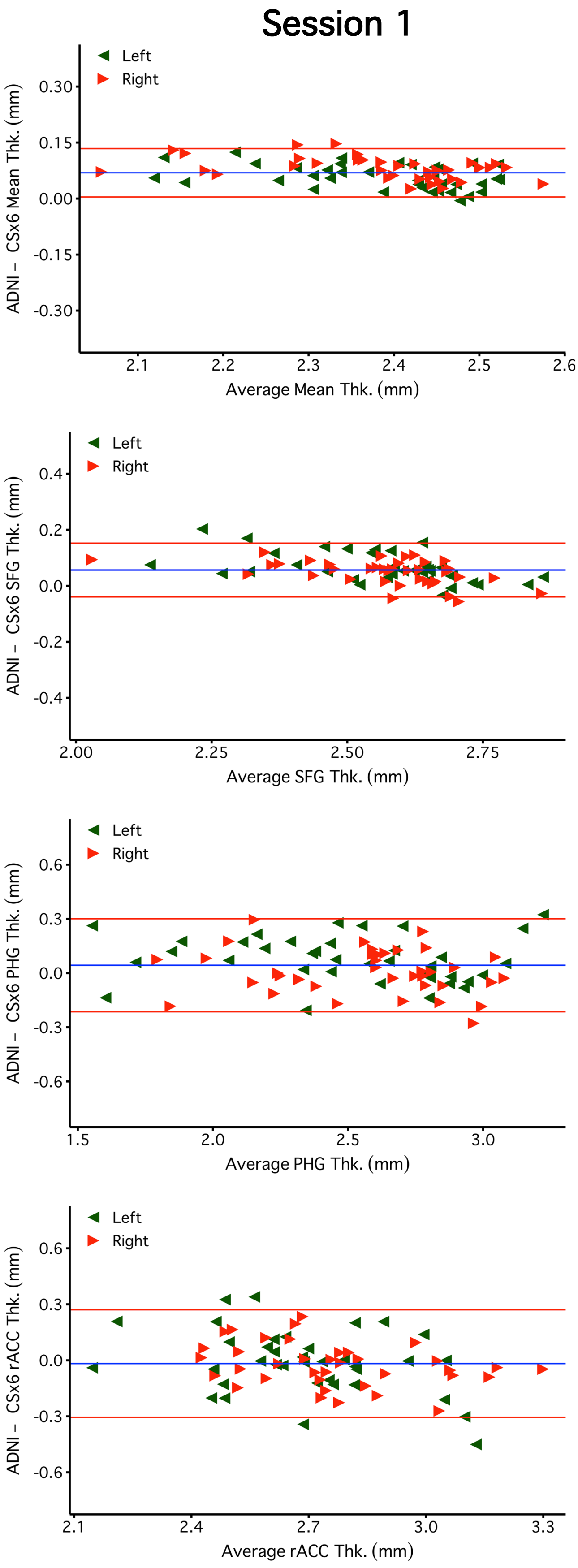

Supplemental Figure 5

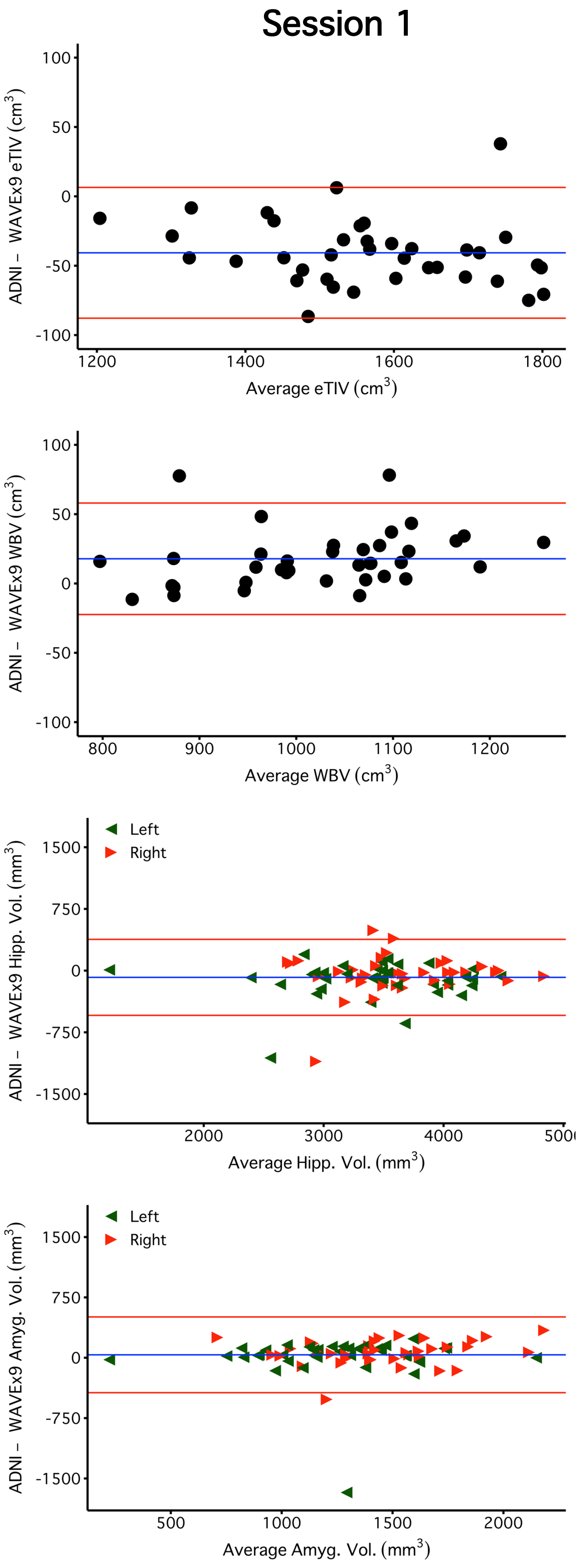

Supplemental Figure 6

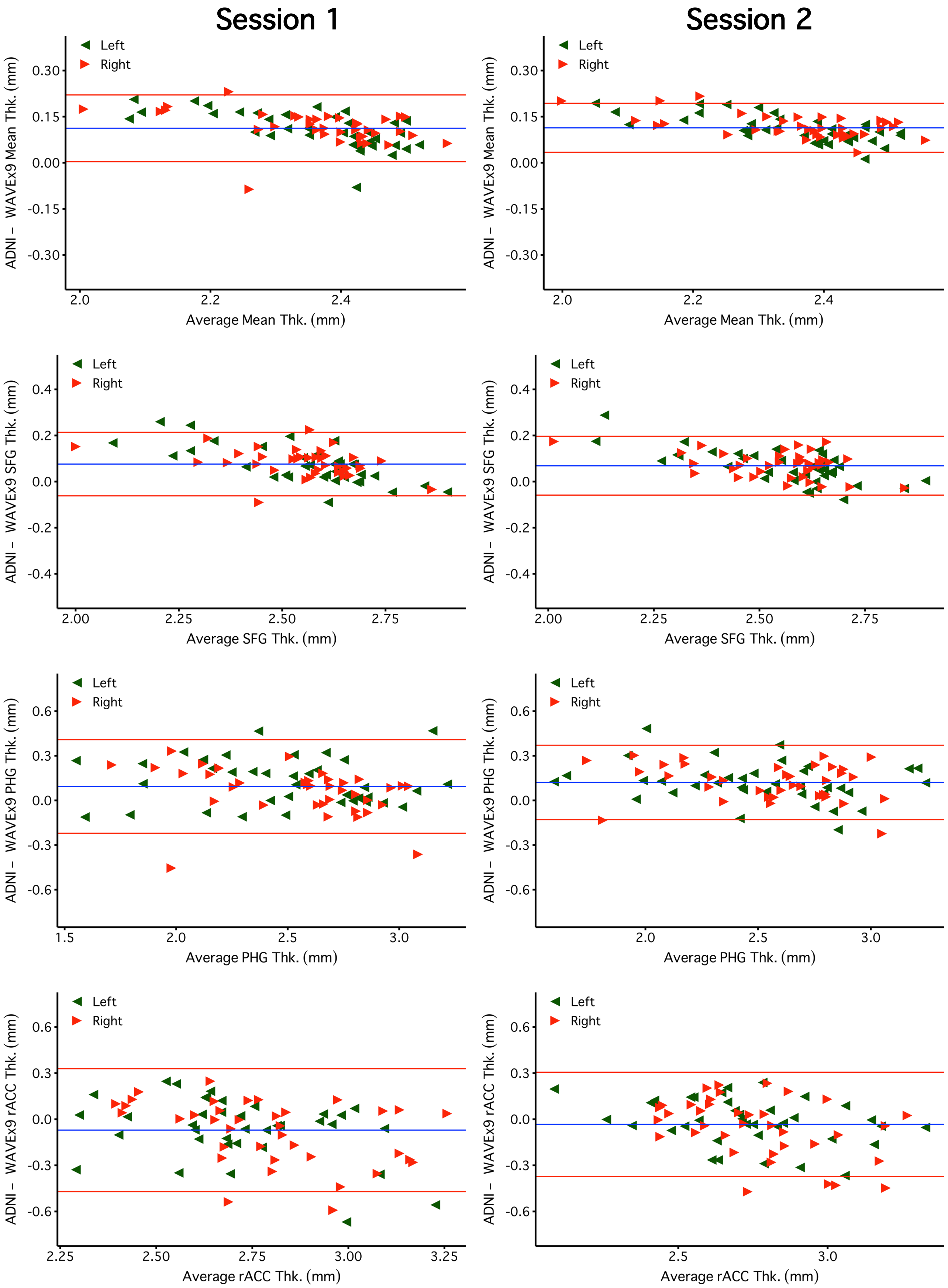

Supplemental Figure 7
