# Supplementary material for: Brain morphometry in older adults with and without dementia using extremely rapid structural scans
Source: Neuroimage. Author manuscript; Available in PMC 2023 Aug 1. (PMC10330834; doi:10.1016/j.neuroimage.2023.120173)
Supplement: 4 [file NIHMS1910689-supplement-4.docx]

**Supplemental Figure 1. Reliability estimates tend to be lower in small regions.** Extending from the data presented in Figures 4 and 5, which illustrate correlation plots for individual measures, the present plots comprehensively show the test-retest correlations (R^2^; y-axis) as a function of size (x-axis) for all measures. The reliability estimates are plotted against the subcortical volume (top, red) or surface area for regional thickness measures (bottom, blue). The volume and surface area for each brain region are obtained from FreeSurfer’s 711_2C atlas. Reliabilities are above R^2^ = 0.75 for most measures. Smaller regions tend to have lower test-retest reliabilities. The pallidum (Pall) has low reliability across scan types and is the second smallest subcortical structure. Several regions in the orbitofrontal cortex have average sizes but low reliabilities indicating consistent estimation challenges across scan types. Additional abbreviations: left (L), right (R), posterior cingulate cortex (pCC), medial orbitofrontal cortex (mOFC), lateral orbitofrontal cortex (lOFC), insula (Ins), rostral anterior cingulate cortex (raCC), peri calcarine (pCalc), lingual (Ling), frontal pole (FP).

**Supplemental Figure 2. Session 2 validity estimated from CSx6 scans are similar to those obtained from the standard ADNI reference scan.** Estimates of convergent validity are displayed as the correlation (R^2^) for each measure displayed in Figures 4 and 5. Plots display the between-subject correlation between brain volume measures estimated from the ADNI images (x-axis) with those estimated from the CSx6 images (CSx6; y-axis). Given each set of scan types was collected over two sessions, two separate R^2^ estimates are available. Session 2 is visualized here and replicates the findings from Session 1 displayed in Figure 9. High correlations are replicable across both sessions and closely cluster along the X = Y identity line, indicating high validity for the extremely rapid CSx6 scans. Note, the values for mean thickness fall off the identity line but remain proportionate across scan types with a high R^2^. This mean shift is likely due to different contrast properties between the ADNI and CSx6 scans leading to a subtle shift in the automated placement of gray/white boundaries that is made clearest in the global measure of mean thickness.

**Supplemental Figure 3. Session 2 measures estimated from WAVEx9 scans are similar to those obtained from the standard ADNI reference scan.** Estimates of convergent validity are displayed as the correlation (R^2^) for each measure displayed in Figures 4 and 5. Plots display the between-subject correlation between brain volume measures estimated from the ADNI images (x-axis) with those estimated from the WAVEx9 images (y-axis). Given each set of scan types was collected over two sessions, two separate R^2^ estimates are available. Session 2 is visualized here and replicates the findings from Session 1 displayed in Figure 10. High correlations are replicable across both sessions and closely cluster along the X = Y identity line, indicating high validity for the extremely rapid WAVEx9 scans. Note, the values for mean thickness fall off the identity line but remain proportionate across scan types with a high R^2^. This mean shift is likely due to different contrast properties between the ADNI and CSx6 scans leading to a subtle shift in the automated placement of gray/white boundaries that is made clearest in the global measure of mean thickness.

**Supplemental Figure 4. Brain volume measures estimated from CSx6 scans show high agreement with those obtained from the standard ADNI reference scan.** Estimates of agreement are displayed using Bland-Altman plots for each measure displayed in Figure 4. Plots display the average size of each morphometric for each participant (x-axis) and the difference between the ADNI and CSx6 estimates for each participant (y-axis). In each plot, the blue horizontal line represents the mean difference between the ADNI and CSx6 estimates across the full sample and the red lines represent 95% confidence intervals (i.e., +/- 1.96 standard deviations) around that mean difference. Given each set of scan types was collected over two sessions, two separate Bland-Altman plots are displayed here in separate columns. Generally, high agreement is found across morphometrics and sessions..

**Supplemental Figure 5. Cortical thickness measures estimated from CSx6 scans show high agreement with those obtained from the standard ADNI reference scan.** Estimates of agreement are displayed using Bland-Altman plots for each measure displayed in Figure 5. Plots display the average size of each morphometric for each participant (x-axis) and the difference between the ADNI and CSx6 estimates for each participant (y-axis). In each plot, the blue horizontal line represents the mean difference between the ADNI and CSx6 estimates across the full sample and the red lines represent 95% confidence intervals (i.e., +/- 1.96 standard deviations) around that mean difference. Given each set of scan types was collected over two sessions, two separate Bland-Altman plots are displayed here in separate columns. Generally, high agreement is found across morphometrics and sessions.

**Supplemental Figure 6. Brain volume measures estimated from WAVEx9 scans show high agreement with those obtained from the standard ADNI reference scan.** Estimates of agreement are displayed using Bland-Altman plots for each measure displayed in Figure 4. Plots display the average size of each morphometric for each participant (x-axis) and the difference between the ADNI and WAVEx9 estimates for each participant (y-axis). In each plot, the blue horizontal line represents the mean difference between the ADNI and CSx6 estimates across the full sample and the red lines represent 95% confidence intervals (i.e., +/- 1.96 standard deviations) around that mean difference. Given each set of scan types was collected over two sessions, two separate Bland-Altman plots are displayed here in separate columns. Generally, high agreement is found across morphometrics and sessions.

**Supplemental Figure 7. Cortical thickness measures estimated from WAVEx9scans show high agreement with those obtained from the standard ADNI reference scan.** Estimates of agreement are displayed using Bland-Altman plots for each measure displayed in Figure 5. Plots display the average size of each morphometric for each participant (x-axis) and the difference between the ADNI and WAVEx9 estimates for each participant (y-axis). In each plot, the blue horizontal line represents the mean difference between the ADNI and CSx6 estimates across the full sample and the red lines represent 95% confidence intervals (i.e., +/- 1.96 standard deviations) around that mean difference. Given each set of scan types was collected over two sessions, two separate Bland-Altman plots are displayed here in separate columns. Generally, high agreement is found across morphometrics and sessions.
